# Role of novel mutations in food vacuole transporters beyond K13-mediated artemisinin resistance in *Plasmodium falciparum*

**DOI:** 10.1128/aac.00293-25

**Published:** 2025-09-30

**Authors:** Iqbal Taliy Junaid, Ashutosh Panda, Arunaditya Deshmukh, Rahila Sardar, Monika Narwal, Prakhar Agrawal, Neha Prakash, Asif Akhtar, Amit Kumar Dey, Suneet Shekhar Singh, Saptarshi Mridha, Jigneshkumar Mochi, Sadaf Parveen, Mohit Kumar, Rashi Nagar, Naseem Gaur, Dinesh Gupta, Asif Mohmmed, Inderjeet Kaur, Krishanpal Karmodiya, Pawan Malhotra

**Affiliations:** 1 Malaria Biology Group, International Centre for Genetic Engineering and Biotechnology (ICGEB) 28845 , New Delhi, India; 2 Translational Bioinformatics Group, International Centre for Genetic Engineering and Biotechnology 28845 , New Delhi, India; 3 Parasite Cell Biology Group, International Centre for Genetic Engineering and Biotechnology (ICGEB) 28845 , New Delhi, India; 4 Department of Biology, Indian Institute of Science Education and Research 193158, Pune, Maharashtra, India; 5 Yeast Biofuel Group, International Centre for Genetic Engineering and Biotechnology 28845 , New Delhi, India; 6 Department of Biotechnology, Central University of Haryana 242287, Mohindergarh, Haryana, India; The Children's Hospital of Philadelphia, Philadelphia, Pennsylvania, USA

**Keywords:** malaria, drug resistance, transporters, mutations, geographical isolates, SNPs

## Abstract

Malaria remains one of the leading causes of morbidity and mortality worldwide, mainly because of the emergence of drug resistance against current antimalarials. The *Plasmodium falciparum* food vacuole (FV) proteins, *P. falciparum* chloroquine (CQ) resistance transporter (PfCRT), PfMDR1 and the cytosolic protein PfKelch13 have been linked to CQ and artemisinin resistance, respectively. Here, we aimed to identify the associations of these resistance markers with mutations in other FV transporters in several field isolates. In this study, we isolated intact *P. falciparum* FVs and carried out detailed proteome analysis to identify new FV transporters. Furthermore, we carried out co-existing mutational analysis for these transport proteins identified in the FV-enriched fraction with known PfKelch13 and PfCRT polymorphisms via single-nucleotide polymorphism (SNP) data from the Pf3K and MalariaGEN databases. Proteome analysis identified 16 transporter proteins in *Plasmodium* FVs. Comparative amino acid analysis of these transporter proteins revealed a coassociation of mutations in several transport proteins identified in the FV-enriched fraction with mutations in the PfKelch13, PfCRT, and PfMDR1 proteins. SNP data analysis of the Pf3K and MalariaGEN databases for 2,517 samples revealed the coassociation of six mutations in four transporter genes, PfCRT, PfNT1, PfCTR2, and PfMDR2, with the PfKelch13 polymorphisms (*P* < 0.0001), suggesting the contribution of additional parasite transporters to the evolution of CQ and artemisinin resistance. Furthermore, functional complementation with the wild-type PfNT1 and PfMFR5 proteins and their mutant forms (PfNT1-F394L, PfMFR5-S278T, and PfMFR5-Y570F) in *Saccharomyces cerevisiae* resulted in resistance to mutant phenotypes in the presence of dihydroartemisinin, suggesting a possible role of these mutations in the acquisition of drug resistance. Together, the genome sequence data from field isolates and yeast complementation analysis of the mutant phenotypes identified novel loci related to PfKelch13-mediated antimalarial resistance and revealed unexplored contributions of transporters to artemisinin resistance.

## INTRODUCTION

Although considerable reductions in malaria morbidity and mortality have been reported over the last two decades, mainly owing to the judicial use of chloroquine (CQ)- and artemisinin (ART)-based combination therapies and other preventive approaches, malaria still caused an estimated 263  million cases and 597 ,000 deaths in 2023 (1). Our failure to completely eradicate malaria has been mainly due to the emergence of parasite resistance to current antimalarials and insecticidal agents. It is therefore essential to develop new drugs as well as an effective malaria vaccine to eradicate malaria (2). In addition, it is critical to understand resistance mechanisms to enable effective surveillance during the deployment of drugs in the field (3, 4).

The *Plasmodium falciparum* food vacuole (FV), also referred to as the digestive vacuole, is a lysosome-like organelle and is the site of profuse metabolic activity, such as hemoglobin (Hb) digestion and hemozoin (HZ) formation, during asexual blood stage development (5, 6). Transcriptomic, proteomic, and interactome studies have shown broad changes in a number of parasite pathways in both CQ- and ART-resistant lines (7–9). Multiple studies have demonstrated that FV metabolic activity, specifically related to Hb endocytosis, proteolysis, and HZ formation, is affected in *P. falciparum* resistance to CQ and ART (3, 4). CQ-resistant parasites exhibit a decrease in CQ accumulation in the FV. The mutant *P. falciparum* CQ resistance transporter (PfCRT) can efflux CQ from the FV away from its site of action, and mutations in PfMDR1 can modulate this level of resistance (4, 10, 11). Similarly, in ART-resistant parasites, mutations in the PfKelch13 protein have been linked with decreased Hb uptake and digestion, resulting in decreased heme production, lower levels of ART activation, and reduced cellular stress (4, 12).

Here, we identified a repertoire of FV membrane transporters by performing liquid chromatography-tandem mass spectrometry (LC‒MS/MS) analysis of purified FVs. Co-occurrence analysis was performed to determine the associations of mutations in the PfKelch13 and PfCRT proteins with mutations in some of the transport proteins identified in the FV-enriched fraction. Additionally, complementation studies in yeast for two previously characterized transport proteins, PfNT1 and PfMFR5, and their mutants provided functional evidence of these mutations in ART resistance. The overall results provide novel insights into the involvement of multiple FV transporters in the broad mechanisms of antimalarial resistance.

## RESULTS

### Proteome analysis of purified *P. falciparum* FVs reveals several transporters

**Fig 1:**
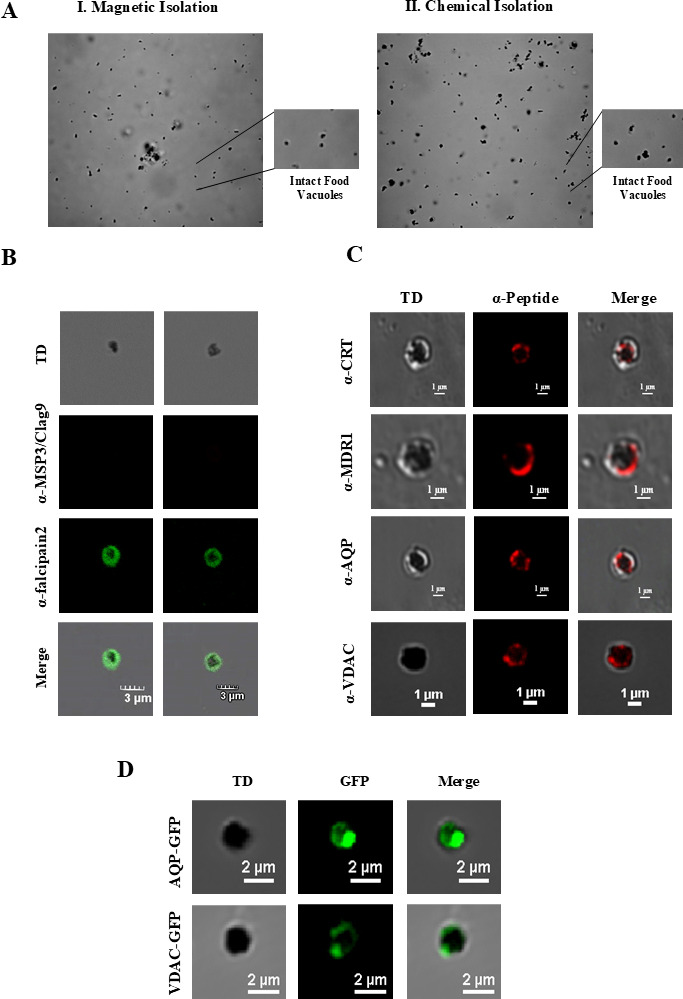
Purity and homogeneity of the FV preparation. (**A**) Confocal images of FVs isolated in I. Magnetic method and II. Chemical method: HZ crystals (dark pigments) are present specifically in FVs. (**B**) Isolated FVs were fixed and immunostained with an anti-falcipain-2 antibody that served as a positive control and with either anti-clag nine or anti-MSP3 antibodies that served as a negative control. (**C**) Immunolocalization studies on isolated FVs using anti-peptide antibodies against PfCRT, PfMDR1, PfAQP, and PfVDAC proteins revealed overlapping expression with HZ crystals (red). (**D**) *P. falciparum* transgenic lines expressing the PfAQP-GFP and PfVDAC-GFPs in isolated FVs, confirming the expression of these transporters in FVs.

FV transporter proteins are central to malaria parasite biology and are key players in the drug resistance mechanisms of *P. falciparum*. In the present study, we applied two independent approaches, magnetic and chemical separation, to purify *P. falciparum* FVs from mature (34–38 hpi) trophozoites. The protocols used for magnetic and chemical purification are described schematically in Fig. S1A and B, respectively. The purity and homogeneity of the preparations were assessed via microscopy, as indicated by the presence of HZ crystals in each FV (Fig. 1A). For all subsequent analyzes, the magnetic isolation method was used due to its higher yield and reproducibility. The purity of FVs was further verified by immunostaining and Western blotting with an anti-Pffalcipain-2 antibody, as falcipain-2 has been shown to localize to the FV (13). Staining with anti-PfMSP3, anti-PfClag9, and anti-PfKAHRP antibodies served as negative controls (Fig. 1B; Fig. S2A and B). In addition, compartment-specific markers were employed to exclude contamination from other cellular organelles. Immunostaining for the nuclear marker histone H3, the plasma membrane protein Niemann-Pick Type C1 (NCR1), and the endoplasmic reticulum (ER) chaperone binding immunoglobulin protein (BiP) showed no detectable signal in FV preparations, indicating minimal contamination from the nucleus, parasite plasma membrane (PPM), or ER (Fig. S3A and B). The purified FVs were subsequently lysed and further processed for LC‒MS/MS analysis.

LC‒MS/MS analysis of purified FVs from *P. falciparum* trophozoites identified 876 and 448 proteins via magnetic and chemical protocol, respectively, from three biological replicates. A total of 418 proteins were commonly detected between the two proteome sets (Fig. S1C; Table S3). Further analysis was performed on the common proteins identified in both the proteome sets.

Importantly, our proteome analysis identified most of the known FV resident and FV membrane proteins, such as falcipain-2, four aspartic proteases (plasmepsins 1-IV), PfCRT, PfMDR1, PfMDR2, and PfV-type H^+^-ATPase (14–18). Since two FV transporters, PfCRT and PfMDR1, have been linked with CQ resistance, we next looked for various transporter proteins in our FV proteome data. On the basis of predicted transporter/TM domain and/or channel properties, a total of 16 transport proteins were identified in the FV proteome data (Table 1). We have also observed the presence of PfKelch 13 protein in our FV data set. Additionally, PfCTR2 has been included in our analysis as it was identified in some of our earlier runs. Alpha-soluble NSF attachment protein (PfSNAP) was included as an intra-FV control in most of the subsequent studies (Fig. S2C).

**TABLE 1:** List of shortlisted membrane/transporter proteins of FVs

Shortlisted proteins				Magnetic				Chemical			
Sl. No.	Accession	Description	MV (kDa)	Coverage (%)	# Peptides	# PSMs	# Unique peptides	Coverage (%)	# Peptides	# PSMs	# Unique peptides
1	PF3D7_1211900	Non-SERCA-type Ca^2+^- transporting P-ATPase	140.2	40	43	256	43	32	35	155	35
2	PF3D7_0523000	Multidrug resistance protein 1	160.2	21	26	119	26	15	18	67	18
3	PF3D7_1447900	Multidrug resistance protein 2	118.9	12	10	58	10	8	6	21	6
4	PF3D7_1343700	Kelch protein K13	83.6	21	10	41	10	14	5	13	5
5	PF3D7_1432100	Voltage-dependent anion- selective channel protein, putative	33.2	17	6	23	6	34	6	17	6
6	PF3D7_1439000	Copper transporter 1	27.1	21	5	10	5	9	1	6	1
7	PF3D7_0103200	Nucleoside transporter 4	50	10	4	20	4	13	4	8	4
8	PF3D7_0824400	Nucleoside transporter 2	67.6	7	4	26	4	6	4	10	4
9	PF3D7_1474600	Vacuole membrane protein 1, putative	46.2	14	4	18	4	3	1	2	1
10	PF3D7_0709000	CQ resistance transporter	48.6	13	3	6	3	7	3	11	3
11	PF3D7_0316600	Formate-nitrite transporter	34.4	9	3	21	3	9	5	18	5
12	PF3D7_1129900	Major facilitator superfamily- related transporter, putative	70.1	6	3	13	3	1	1	5	1
13	PF3D7_1223700	Vacuolar iron transporter	31	7	3	14	3	4	1	2	1
14	PF3D7_1132800	Aquaglyceroporin	28.3	10	2	8	2	7	1	4	1
15	PF3D7_1116500	Folate transporter 2	51.3	3	1	10	1	3	1	12	1
16	PF3D7_1347200	Nucleoside transporter 1	47.6	3	1	3	1	4	3	13	3

### Immunostaining and immunoblot analysis of 3D7 and transgenic lines confirmed the presence of these new transporters in *P. falciparum* FVs

**Fig 2:**
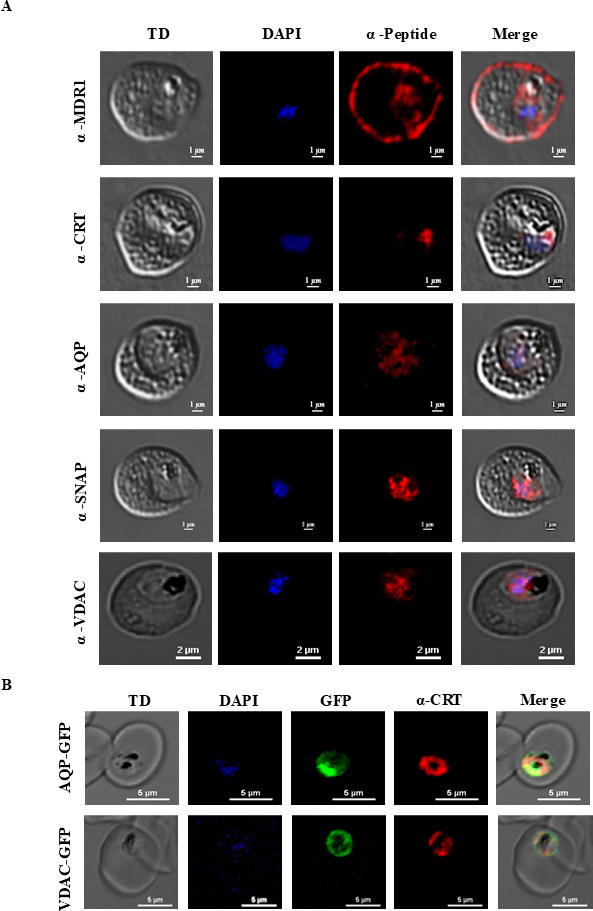
Immunofluorescence analysis of *P. falciparum* trophozoites confirmed the expression of PfMDR1, PfCRT, PfAQP, PfSNAP, and PfVDAC in *Plasmodium* FVs. (**A**) Immunostaining was performed for fixed *P. falciparum* trophozoites via anti-peptide PfMDR1, PfCRT, PfAQP, PfSNAP, and PfVDAC antibodies. These antibodies revealed that the expression of these proteins in FVs overlapped with that in the HZ crystals. (**B**) *P. falciparum* transgenic lines expressing the PfAQP-GFP and PfVDAC-GFP confirmed the expression of the PfAQP and PfVDAC proteins in FVs at the trophozoite stage.

To determine the expression of some of the transport proteins identified in the FV-enriched fraction, we generated peptide-specific antibodies against five proteins and generated two transgenic lines expressing the PfAQP-green fluorescent protein (GFP) and PfVDAC-GFP. Immunolocalization studies on *P. falciparum* trophozoites as well as on purified FVs using anti-PfCRT, anti-PfMDR1, anti-PfAQP, anti-PfVDAC, and anti-PfSNAP antibodies revealed the expression of these proteins in FVs overlapping the HZ crystals (Fig. 1C and 2A; Fig. S2C). These results were confirmed by the expression of the PfAQP-GFP and PfVDAC-GFP fusion proteins in FVs (Fig. 1D and 2B; Fig. S5). Immunoblot analysis of the purified FV extract further validated the expression of five of the identified proteins in the *P. falciparum* FV (Fig. S2D).

### Linking mutations in several FV transporter proteins with CQ or ART resistance via published genome sequences

A comparative amino acid sequence analysis for all the identified *P. falciparum* FV transporters was subsequently performed via published genome sequences of the *P. falciparum* strains 3D7, Dd2 (CQ-, pyrimethamine-, and mefloquine-resistant strain), 7G8 (CQ- and pyrimethamine-resistant strain), GB4 (CQ-resistant strain), and KH02 (ART-resistant strain) in the PlasmoDB database. Table 2 describes the comprehensive details of various mutations in FV transporter proteins along with mutations in PfKelch13 transporter protein in these strains. Several mutations in transport proteins identified in the FV-enriched fraction were observed, in addition to the reported mutations in PfCRT, PfMDR1, and PfKelch13 transporter proteins in these different *Plasmodium* strains (Fig. S8). The PfKelch13 protein presented K189T and C380Y mutations in the GB4 and KH02 parasite lines, respectively; however, it did not present mutations in the Dd2 and 7G8 strains (Table 2). Given that little is known about mutations in transporter proteins in ART-resistant lines, we next performed mutational analysis for nine of the transport proteins identified in the FV-enriched fraction, PfMDR1, PfMDR2, PfNT1, PfNT4, PfMFR5, PfATP4, PfCTR2, PfAQP, and PfUGT, via PCR amplification and sequencing via different sets of primers targeting the extracellular or transmembrane (TM) domains of two drug-resistant lines, IPC 5202 (MRA1240) and INDO (MRA-819), whose genome sequences are not available (Fig. S6 and 7). The IPC5202 and INDO strains are resistant to ART and CQ, respectively. Three independent sequence analyses were conducted for each PCR fragment, and the fragments were sequenced in full on both strands. Mutations in seven of these transport proteins were identified in these drug-resistant lines (Table 2). Although the roles of these transporter proteins in antimalarial drug resistance need further validation via genetic manipulation and functional assays, sequence analysis suggests that mutations in these transporters may contribute broadly to antimalarial resistance phenotypes.

**TABLE 2:** Mutation analysis of the shortlisted proteins among various strains of *P. falciparum^a^*

S. no	Plasmodb id	GENE	Protein name	Reported mutations	DD2/3D7	7G8/3D7	GB4	KH02	INPO (MRA-819) strain	IPC 5202 (MRA1240) strain
1	PF3D7_1211900	ATP4	Non-SERCA-type Ca^2+^ -transporting P-ATPase		G1128R	E30G, S86N, Q1081K, G1128R, I1237V	Q1081K, G1128R	G1128R	No change	No change
2	PF3D7_0523000	MDR1	Multidrug resistance protein 1	N86Y, Y184F, N661del, S1034C, N1042D, D1246Y (19)	N86F, N662del	Y184F, S1034C, N1042D, N661K, D1246Y	N86Y, Y184F, D650N, N652D	Y184F, N659DEL, N660DEL, N661DEL, S784L,	Y184F, S1034C, N1042D	Y184F
3	PF3D7_1447900	MDR2	Multidrug resistance protein 2	S208N, G299D, F423Y, T484I, I492V (20)	N318del, S208N, G299D, F423Y, T484I	S208N, F423Y, I492V	N318del	S208N, G299D, N318del, F423Y, T484I	No change	T484I
4	PF3D7_1343700	Kelch13	Kelch protein K13	F446I, N458Y, M476I, Y493H, R539T, I543T, P553L, R561H, P574L, C580Y (21)	No change	No change	K189T	C580Y	NP	NP
5	PF3D7_1432100	VDAC	Voltage-dependent anion-selective channel protein, putative		No change	No change	No change	No change	NP	NP
6	PF3D7_1439000	CTR1	Copper transporter 1		S211L	No change	No change	No change	NP	NP
7	PF3D7_0103200	NT4	Nucleoside transporter 4		S383A	N209I	No change	S383A	S383A	S383A
8	PF3D7_0824400	NT2	Nucleoside transporter 2		S308T,S424T,	N53K, S308T, S424A, L470F, L524F, N525Y	No change	S308T, S424T	NP	NP
9	PF3D7_1474600	VMP1	Vacuole membrane protein 1, putative		No change	No change	No change	No change	NP	NP
10	PF3D7_0709000	CRT	CQ resistance transporter	C72S, M74I, N75E, K76T, A220S, Q271E, N326S, N326D, I356T, R371I (10)	M74I, N75E, K76T, A220S, Q271E, N326S, I356T, R371I	C72S, K76T, A220S, N326D, I356L	M74I, N75E, K76T, A220S, Q271E, R371I	M74I, N75E, K76T, A220S, Q271E, N326S, I356T, R371I	NP	NP
11	PF3D7_0316600	FNT	Formate-nitrite transporter		H159D	No change	H159D	H159D, E283G	NP	NP
12	PF3D7_1129900	MFR5	Major facilitator superfamily -related transporter, putative		No change	T119A, S278T, Y570F	A606V	No change	No change	^*b*^V549M
13	PF3D7_1223700	VIT	Vacuolar iron transporter		No change	No change	No change	No change	NP	NP
14	PF3D7_1132800	AQP	Aquaglyceroporin		P130S	No change	No change	P130S	P130S	No change
15	PF3D7_1116500	FT2	Folate transporter 2		No change	No change	No change	No change	NP	NP
16	PF3D7_1347200	NT1	Nucleoside transporter 1	Q284E, F394L (22)	F394L	Q284E	No change	F394L	F394L	F394L
17	PF3D7_1421900	CTR2	Copper transporter 2, putative		E49G	No change	No change	E49G	E49G	No change
18	PF3D7_1113300	UGT	UDP-galactose transporter, putative	F37V (23)	No change	No change	No change	No change	No change	No change

^*a*^ NP, Not performed.

^*b*^ Novel mutation.

### Prevalence of the identified mutations in the field isolates and their co-existence with PfKelch13 polymorphisms

**Fig 3:**
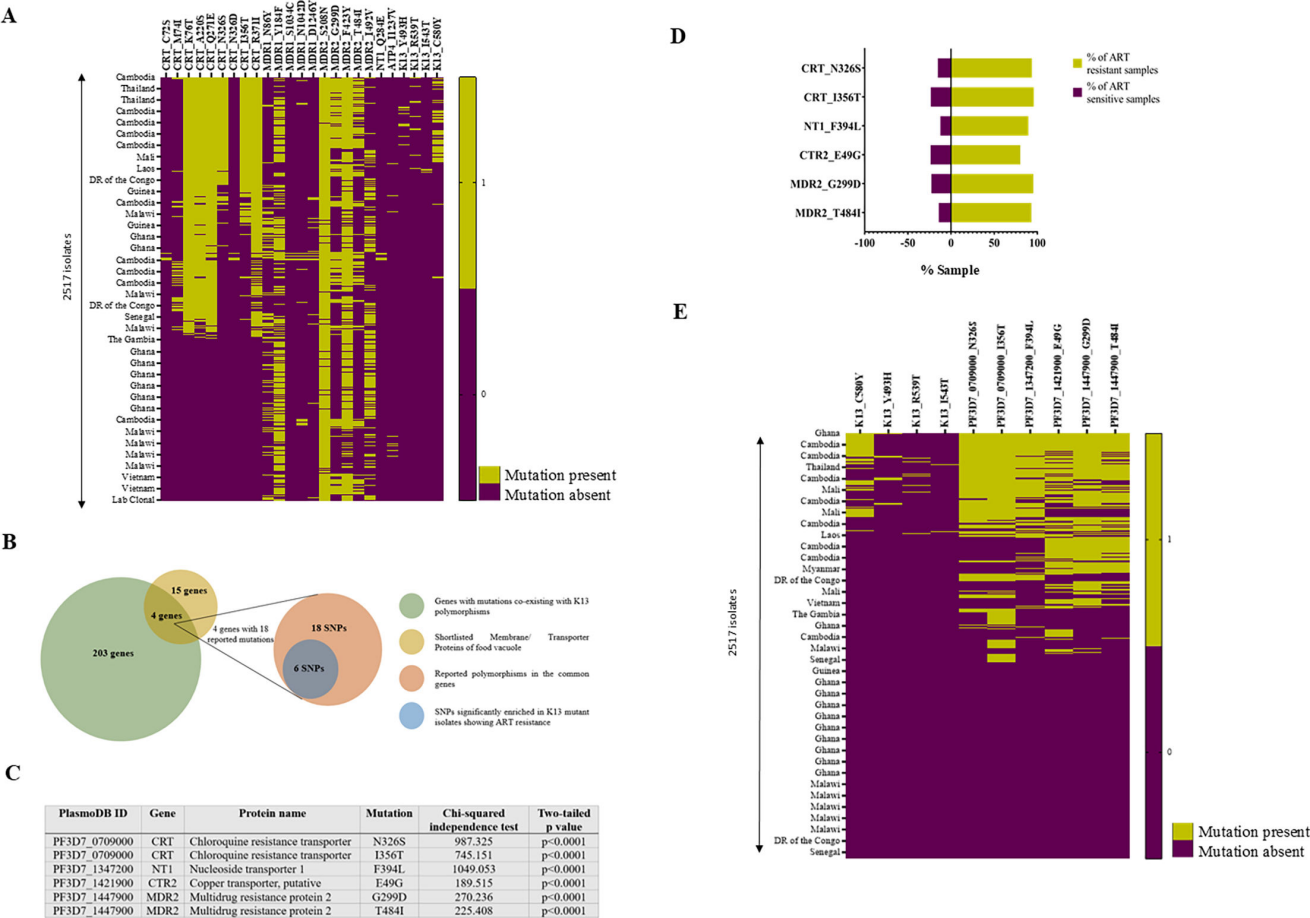
Prevalence of the identified mutations in the field isolates and their co-existence with PfKelch13 polymorphisms. (**A**) Plot showing co-existence of all the mutations shortlisted in the genome data set of 2,517 field isolates of *P. falciparum*. Ochre color represents the presence of the mutation in an isolate, while magenta represents the absence of the same. (**B**) Common genes having mutations co-occurring with PfKelch13 mutations (C580Y, R539T, and Y493H) and genes of FV. Out of the 207 genes (green circle) with mutations enriched along with K13 SNPs, four were found to be common with the list of FV transporter genes shortlisted in this study. From these four genes, a total of 18 mutations were reported (orange circle), six of which were found to be significantly enriched along with K13 mutations in ART-resistant isolates (blue circle). (**C**) List of SNPs that were significantly enriched along with K13 mutant isolates along with their χ test statistics. (**D**) Presence of the above-mentioned polymorphisms in ART-resistant (harboring K13 mutations) and ART-sensitive (without K13 mutations) isolates. (**E**) Heatmap to signify the co-occurrence patterns of the abovementioned SNPs along with K13 mutations, which confer ART resistance. Ochre color represents the presence of the mutation in an isolate, while magenta represents the absence of the same.

Next, we analyzed the status of the mutations identified in the FV transporter proteins in the field isolates by reanalyzing the publicly available whole-genome sequencing data procured from Pf3K, MalariaGEN, which consists of 2,517 samples from 15 countries in Southeast Asia and Africa (24). Variant annotations for the data were performed via snpEff version 4.3 (25), and subsequently, a binary matrix was created, with 0 indicating the absence of a mutation and one representing the presence of the same mutation. From this matrix, our mutations of interest were filtered out and checked for their co-existence patterns. According to the heatmap (Fig. 3A), the prevalent CRT mutations, such as K76T, A220S, Q271E, N326S, I356T, and R371I, strongly co-exist with the MDR mutations Y184F, S208N, G299D, F423Y, and T484I.

To explore the presence of mutations in FV transporter proteins in ART-resistant parasites, we investigated their co-occurrence with PfKelch13 mutations (a definitive marker of ART resistance) in the abovementioned field isolates. Although PfKelch13 mutations are not widely prevalent among these field isolates, we found that four of the transporter genes (PfCRT, PfNT1, PfCTR2, and PfMDR2) have mutations enriched along with PfKelch13 mutations (Fig. 3B and E). In these four genes, a total of 18 polymorphisms (single-nucleotide polymorphisms [SNPs]) were reported, of which six SNPs were specifically enriched in the ART-resistant parasites (Fig. 3B). To statistically examine the co-occurring mutations in transporter genes and PfKelch13, a χ test of independence was performed. A 2 × 2 grid format was used to compare the combination of wild-type (WT)/mutant instances of K13 with WT/mutant instances of the candidate genes (Table S1). Interestingly, all six mutations in transporter genes were significantly associated with the K13 polymorphisms, with *P* < 0.0001 (Fig. 3C). A global distribution profile of PfKelch13 and transporter gene mutations (Fig. 3D and E) further revealed an enrichment of transporter protein mutations in ART-resistant field isolates. Taken together, our analysis revealed co-occurrence and co-association between PfKelch13 mutations and transporter protein mutations in field isolates of *P. falciparum* and revealed the involvement of transporter protein mutations in both CQ- and ART-resistant parasites.

### Site-directed mutagenesis and yeast complementation assays reveal a role for PfNT1 and/or PfMFR5 in conferring resistance to *P. falciparum* against DHA

**Fig 4:**
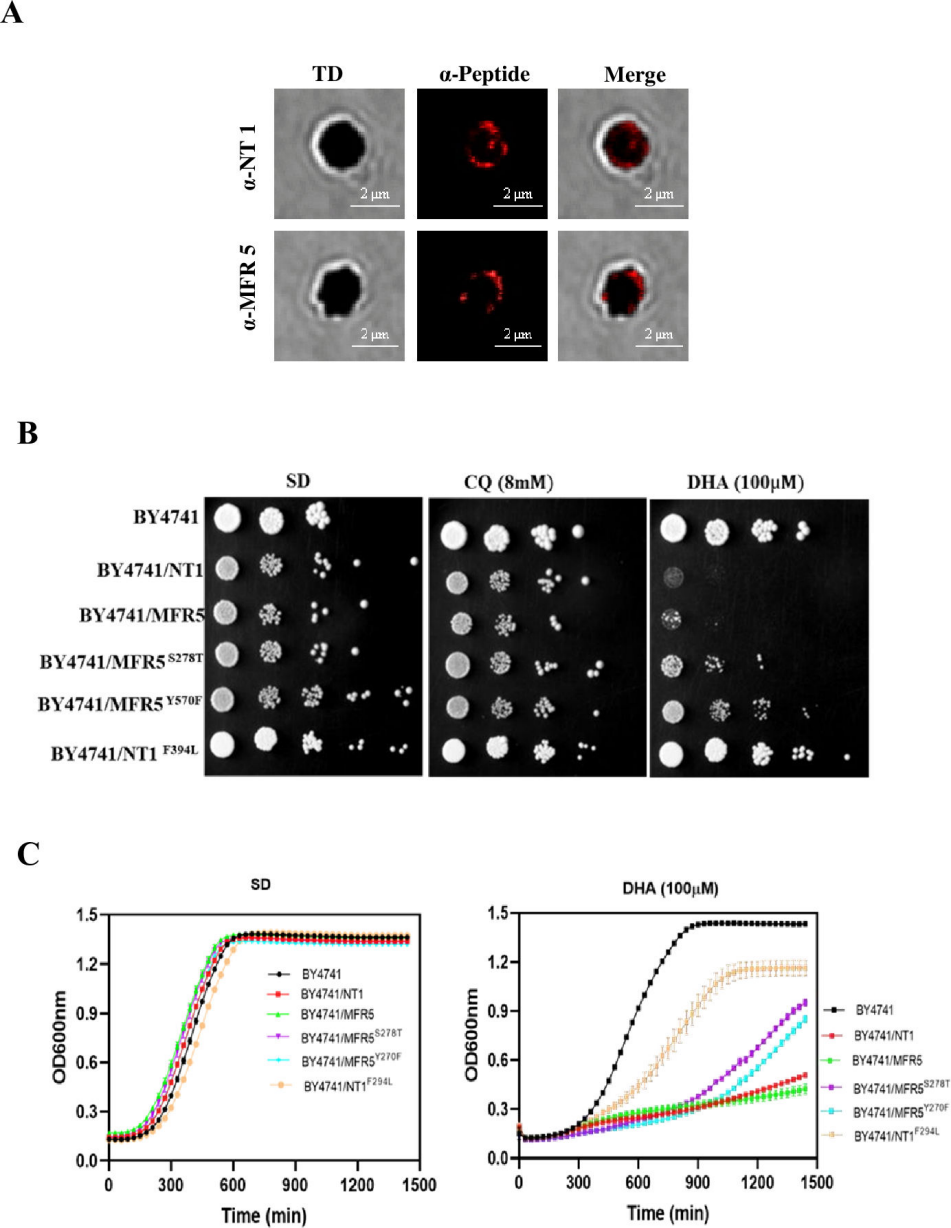
Complementation of PfNT1 and PfMFR5 in a yeast orthologue system and the effects of CQ and DHA on mutant yeast strains. (**A**) Immunolocalization studies on isolated FVs using anti-peptide antibodies against PfNT1 and PfMFR5 proteins revealed overlapping expression with HZ crystals (red) further confirming the localization of these proteins in FV. (**B**) Spot assay showing the effects of the compounds CQ (8 mM) and DHA (100 µM) on the different yeast lines. Compared with those treated with CQ, WT PfNT1 or PfMFR5 proteins were highly susceptible to DHA treatment. The mutants reversed the sensitivity to DHA in the case of PfNT1-F3934, PfMFR5-S278T, and PfMFR5-Y570F. (**C**) Time-dependent growth assay to confirm the role of mutations in DHA susceptibility and the experiments were performed in biological triplicates (*n* = 3).

To elucidate the role of FV-localized transporters in antimalarial resistance, we performed yeast complementation assays using *Saccharomyces cerevisiae* BY4741 expressing either WT or mutant versions of PfNT1 and PfMFR5. The subcellular localization of these transporters to the FV was confirmed by immunofluorescence staining of isolated *P. falciparum* FVs and trophozoites using peptide-specific antibodies (Fig. 4A; Fig. S3C).

The codon-optimized genes encoding WT PfNT1 or PfMFR5 proteins were cloned and inserted into pGPD2 for functional expression in *S. cerevisiae BY4741*. Site-directed mutagenesis was performed to introduce targeted mutations into the *PfNT1* and *PfMFR5* genes to investigate the role of mutations in the PfNT1 and PfMFR5 proteins. For PfNT1, a single mutation, F394L, was introduced, substituting phenylalanine with leucine. In the case of the PfMFR5 protein, two mutations were introduced: S278T serine was substituted with threonine and Y570F, where tyrosine was replaced with phenylalanine, as discussed in the Materials and Methods section.

*S. cerevisiae BY4741* was subsequently transformed with either the WT or mutant *PfNT1* or *PfMFR5* gene. The transformed yeasts were subjected to phenotypic assays to assess their growth and antimalarial drug susceptibility profiles. The spotting and growth assays were performed with and without the antimalarial drugs CQ and dihydroartemisinin (DHA) at concentrations of 8 mM and 100 µM, respectively.

As shown in Fig. 4B, yeast cells expressing WT PfNT1 or PfMFR5 proteins were more susceptible to DHA treatment than were those treated with CQ. The WT yeast strain showed no significant susceptibility to either CQ or DHA. Interestingly, the F394L mutation in the PfNT1 protein reversed the susceptibility of yeast to DHA as well as to CQ. Similarly, yeast expressing the PfMFR5 protein with either the S278T or Y570F mutation presented a noticeable reversal in DHA susceptibility, indicating that this protein is involved in the transport of DHA and that these mutations may contribute to DHA resistance. This phenotype was corroborated by growth kinetics (Fig. 4C), where the WT transporter strains showed pronounced growth inhibition in the presence of DHA, while mutant strains had significantly improved growth over time. Together, these findings, along with the prevailing mutations in ART-resistant parasites in different geographical isolates, suggest that PfNT1 and PfMFR5 could function as transporters involved in the uptake or efflux of DHA and that the specific mutations within these proteins can alter their sensitivity to DHA.

### *In silico* structural analysis provides a robust platform for understanding the effects of mutations on ART resistance

**Fig 5:**
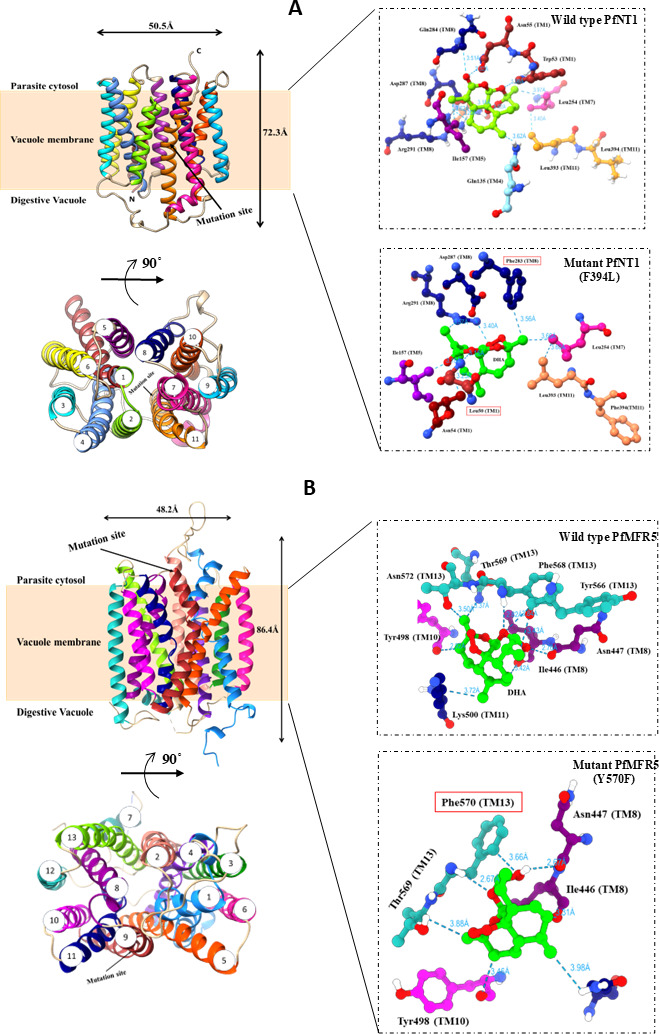
Mapping of drug resistance mutations onto the modeled structures of PfNT1 and PfMFR5 proteins. (**A**) Homology model of PfNT1 predicted 11 TM helices localized within the FV membrane of *P. falciparum*. The right panel shows PfNT1-DHA interactions in the WT and mutant structures. DHA ring moieties adopted a different orientation in mutant as compared to the WT PfNT1. (**B**) Homology model of PfMFR5 predicted 13 TM helices localized within the FV membrane of *P. falciparum*. The right panel shows PfMFR5-DHA interactions in the WT and mutant structures. The mutant protein shows a reduced binding of DHA molecule due to rotamer shift within the binding site. The residues from WT and mutants are shown as sticks and are colored as per the helix of TM

To further understand the effects of point mutations on drug resistance in two of these transporter proteins, PfNT1 and PfMFR5, *in silico* homology modeling via the i-TASSER web server was performed. A total of five models each (PfNT1 and PfMFR5) from I-TASSER (Threading) were generated. We selected only the best model with the lowest *C*-score for further analysis. The *C*-scores for the models PfNT1 (−0.96) and PfMFR5 (−1.76) were well within acceptable limits. An assessment of their stereo quality via a Ramachandran plot revealed that PfNT1 and PfMFR5 had 92% and 85% residues within the allowed regions, respectively. The model predicted a monomeric form of PfNT1 consisting of 11 TM alpha helices spanning two segments/regions (TM1-TM6, and TM7-TM11), together forming a central ligand binding cavity. Similarly, the PfMFR5 model predicted 13 TM alpha helices spanning the lipid bilayer (Fig. 5A and B). Structural analysis revealed that the mutation in PfNT1 (F394L) is located at TM11 and that the mutation in PfMFR5 (Y570F) is located at TM13. Molecular docking of DHA with the modeled WT and mutant structures of PfNT1 and PfMFR5 further revealed that the DHA ring moieties caused a conformational change in the mutant PfNT1 protein compared with the WT PfNT1 protein. However, no such conformational changes were observed in mutant PfMFR5 in the presence of DHA (Fig. 5A and B).

Additionally, we observed that the DHA molecule forms eight interactions in WT PfNT1; however, in mutant PfNT1, two key interactions (Leu50 and Phe283) were disrupted, further reducing the stability of the DHA interaction by altering the atomic conformations within the cavity (Fig. 5A). Additionally, we calculated the electrostatic potential surfaces of the central cavity for WT and mutants PfTN1 and PfMFR5. Compared with the mutant PfNT1, the WT PfNT1 exhibited the greatest electronegativity in the central cavity, which is consistent with the highest affinity for the DHA molecule (Fig. S9A and B). Similarly, docking studies of the DHA molecule with WT and mutant PfMRF5 revealed that the mutant protein shows reduced binding of the DHA molecule due to a rotamer shift within the binding site, thus weakening the interaction with the DHA molecule (Fig. 5B). Together, the modeling data indicate that the WT PfNT1 and PfMRF5 proteins help retain DHA within FVs by interacting with DHA. These mutations weaken the interactions between these transporters and DHA, thereby reducing the retention of DHA in FVs and increasing the efflux of DHA.

## DISCUSSION

Understanding the molecular mechanisms of drug resistance is critical for the deployment and effectiveness of combination therapies in the field to combat malaria parasites (4, 26). *P. falciparum* FV is the target of many antimalarials, and major mechanisms of resistance include either point mutations in FV membrane proteins that mediate drug transport or point mutations in proteins that bind these drugs (3, 12, 27). In the case of CQ-resistant parasites, mechanistic studies have provided evidence that single-nucleotide variations (SNVs) in two transporter proteins, PfCRT (C72S, M74I, N75E, K76T, A220S, Q271E, N326S, N326D, I356T, and R371I) and PfMDR1 (N86Y, Y184F, N661del, S1034C, N1042D, and D1246Y), are primarily responsible for decreased accumulation of CQ in the FV because of energy-dependent drug efflux mechanisms (10, 19, 28, 29). ART resistance in *P. falciparum* is linked mainly to SNVs (F446I, N458Y, M476I, Y493H, R539T, I543T, P553L, R561H, P574L, or C580Y) in PfKelch13, which appear to regulate the rate of endocytosis of Hb within FVs and exert other physiological effects that reduce ART toxicity (12, 21, 30, 31). A recent study by Bhattacharjee et al. (32) has shown the presence of GFP-tagged and native PfKelch13 on the membranous compartments proximal to the parasite FV and to cytosomal structures near the parasite periphery, further supporting the association of PfKelch13 with FV.

Recent studies using gene-edited, isogenic *pfcrt-*modified mutant parasite lines have demonstrated geographical region-specific parasite susceptibility to multiple antimalarial drugs, whose mode of action intersects with Hb import and heme detoxification (33). Transporter-mediated homeostasis of metabolites, lipids, and ions is key for the functionality of the *Plasmodium* FV. To date, only a few transporters are known to be localized/active at the FV membrane (34). In the present study, we undertook a detailed proteomic analysis of purified *P. falciparum* FVs to understand the spectrum of FV transport proteins and analyze their sequences in CQ, ART, and other multidrug-resistant lines in detail. Proteome analysis of *P. falciparum* FVs purified by two independent isolation procedures identified 418 proteins in common, which was approximately four times greater than the 116 previously reported FV proteins (35). This could be attributed to the type of mass spectrometer utilized in both the studies. The present study has utilized an advanced mass spectrometer (Thermo Scientific Orbitrap Fusion Lumos Tribrid). The architecture of this instrument includes a quadrupole, an Orbitrap, and an Ion Trap mass analyzer, which allows for high acquisition rates and resolution of ions. The study by Lamarque et al. (35) was performed on Bruker Daltonics Esquire 3000 Plus Mass Spectrometer, which is now an obsolete model. We could identify 86 out of 116 previously reported proteins in our proteome analysis (Table S4). Careful analysis of transport proteins identified 16 of these proteins in the FV proteome. Transporter-mediated homeostasis of ions, metabolites, and lipids is key for the functionality of the *Plasmodium* FV. However, to date, only a few transport proteins are identified on FV membrane including proton pumps (36), metabolite transporter superfamily members (37), ammonium bicarbonate (ABC) transporters (38), and formate-nitrite transporter (39, 40). In the present study, we further identified a few transporter proteins with proven localization on FV membrane.

Isolating FVs from *Plasmodium* is technically challenging due to their close association with other organelles and the parasite’s small size. Despite employing detergent lysis, mechanical disruption, and differential centrifugation, complete separation from the PPM, ER, and nucleus is difficult. To assess potential contamination, we performed immunostaining with compartment-specific markers, confirming the absence of nuclear (histone H3), PPM (NCR1), and ER (BiP) proteins. However, trace impurities from other cellular compartments may still persist due to the inherent limitations of FV purification.

To explore the potential associations between some of these identified FV transporters and antimalarial resistance, published genome data of four established drug-resistant parasite lines, Dd2 (multidrug-resistant), 7G8 (multidrug-resistant), GB4 (CQ-resistant), and KH02 (ART-resistant), were analyzed and compared with the genomic data of the 3D7 line. Comparative genomic data revealed mutations in many of these FV transporter proteins (Table 2). A similar approach was previously applied for the *pfcrt* and *pfmdr1* genes to identify sensitive or resistant *P. falciparum* lines among various field isolates (10, 41). Few mutations in transport proteins identified in the FV-enriched fraction (PfMDR1, PfMDR2, PfNT1, PfNT4, PfMFR5, PfATP4, PfCTR2, PfAQP, and PfUGT) were confirmed by PCR amplification and sequencing of the extracellular and TM domains in the INDO (CQ-resistant) and IPC 5202 (ART-resistant) strains and were compared with the available genome data set.

We further analyzed the mutations in these transporter proteins in 2,517 field isolates obtained from WGS data (Pf3k and MalariaGEN project). The CRT mutations were K76T, A220S, Q271E, N326S, I356T, and R371I; the MDR mutations Y184F, S208N, G299D, F423Y, and T484I were highly prevalent among samples from Southeast Asia and Africa. These mutations were found to have a high level of co-existence, suggesting that the selective evolution and accumulation of FV protein mutations is a generic way to develop resistance against antimalarials adopted by malaria parasites. Even in the case of ART-resistant parasites (with Y493H, R539T, I543T, or C580Y mutations in the PfKelch13), a strong correlation was observed for the presence of mutations in other FV proteins. The CRT mutations N326S and I356T, the NT1 mutation F394L, the CTR2 mutation E49G, and the MDR2 mutations G299D and T484I were observed to be specifically enriched in ART-resistant parasites and absent in ART-sensitive parasites. The hypothesis that additional determinants can contribute to multidrug resistance is consistent with the earlier finding of an association between ART resistance and nonsynonymous mutations in *fd* (ferrodoxin), *arps10* (apicoplast ribosomal protein S10), *pfmdr2*, and *pfcrt*, which may provide a genetic background that augments the role of mutant K13 as the primary determinant (20). In addition, a recent study revealed that polymorphisms in *Pfmdr1* and *Pfcrt* result in the redistribution of drugs between FVs and the cytosol; this change is the key driver of antimalarial resistance, variability between multidrug resistance phenotypes, and collateral drug sensitivities in these parasites (42).

To further illustrate the effect of newly identified mutation(s) on antimalarial resistance, complementation studies were performed in yeast cells (*S. cerevisiae*) by transforming *S. cerevisiae* with WT or mutant PfNT1 and PfMFR5 proteins. Yeast expressing WT or mutant PfNT1-F394L proteins and WT PfMFR5 or its mutants, S278T and Y570F proteins, were treated with various concentrations of CQ and DHA. The growth curve revealed a greater growth pattern in yeast expressing the mutant PfNT1-F394L protein than in yeast expressing WT PfNT1 in the presence of DHA. Additionally, docking studies with DHA further strengthened our findings, suggesting the possible involvement of mutations in the PfNT1 and PfMFR5 proteins in resistance to DHA. The results presented in this study indicate that many FV transport proteins play important roles in the transport or retention of drugs within FVs and that mutations in some of these transporters change the dynamics of drug retention within the FV. Although the present study only performed yeast complementation assays for two of the transporter proteins, PfNT1 and PfMFR5, the role(s) of other transporters need to be validated by either yeast complementation assays or by gene editing studies in *P. falciparum* lines. The data presented here nevertheless support more multicentric genome-wide associations among the various transporters along with gene-editing studies to further test the co-contributions of various transporters to antimalarial drug resistance.

## MATERIALS AND METHODS

### *P. falciparum* culture and isolation of *Plasmodium* FVs

The *P. falciparum* parasite line 3D7 was maintained and synchronized as described elsewhere (43). For the isolation of FVs, *P. falciparum* parasites were harvested, and FVs were purified via chemical and magnetic column isolation. For chemical isolation, we followed protocols described elsewhere (35). For the magnetic column isolation of *P. falciparum* FVs, the late-stage trophozoites were harvested by saponin lysis. The harvested trophozoites were lysed in ice-cold water pH 4.5 and triturated through a 27G needle followed by DNase I treatment. The lysate was centrifuged at 13,000 rpm for 10 mins. The pellet was resuspended in Buffer M (pH 7.4, containing 2 mM MgSO_4_, 100 mM KCl, 10 mM NaCl, 25 mM HEPES, 25 mM NaHCO_3_, and 5 mM sodium phosphate) and was loaded on LS column (Miltenyi Biotec, Germany). The column was subjected to a MACS separator allowing the lysate to pass with the help of gravity followed by washing twice with phosphate-buffered saline (PBS). The isolation of magnetically associated organelles was further carried out by removing the column from the magnetic separator and eluting the bound vesicles with 2 to 3 mL of PBS via the plunger supplied with the column. The eluted FV was then washed twice with PBS.

### *P. falciparum* parasite transfection

To generate a GFP-tagged transfection vector construct, the entire open reading frames of PfAQP and PfVDAC were amplified via gene-specific primers (Table S2) and cloned and inserted into the pSSPF2 vector at the *Bgl*II and *Avr*II restriction sites to create a fusion of the desired gene of interest with GFP under the control of the *hsp86* promoter (Fig. S4A and B) (43). Synchronized *P. falciparum* 3D7 ring-stage parasites were transfected with 100 µg of purified plasmid DNA by electroporation (310 V, 950 µF), and the transfected parasites were selected with 2.5 µg/mL blasticidin (43).

### Isolation of FV proteins, trypsin digestion, and LC‒MS/MS analysis

FV preparations isolated via both methods were subjected to lysis in radio-immunoprecipitation assay buffer (RIPA) buffer. The lysate was treated with 10% trichloroacetic acid, 5% acetone, and 5 mM dithiothreitol (DTT). The samples were air-dried and resuspended in 100 mM ABC buffer containing 8 M urea. The resuspended solution was diluted with 100 mM ABC buffer to make it 1 M urea, followed by in-solution trypsin digestion. The FV samples were first reduced with 10 mM DTT (final concentration) for 1 h at room temperature (RT) . After reduction, the mixture was alkylated with 40 mM iodoacetamide (Sigma‒Aldrich, USA) for 1 h at RT in the dark. Proteins were digested by the addition of trypsin at a ratio of 1:50 (wt/wt) (trypsin:protein) at 37°C for 12‒16 h. After digestion, the extracted peptides were acidified to 0.1% formic acid and analyzed by an Orbitrap Fusion Lumos mass spectrometer coupled with a Nano-LC 1200 instrument (Thermo Fisher Scientific, Inc., USA). The raw data were analyzed via Proteome Discoverer, version 2.4, via the SEQUEST and AMANDA algorithm. Protter Software Version 1 (44) was used for the integration and visualization of both annotated and predicted protein sequence features. The mass spectrometry proteomics data have been deposited to the ProteomeXchange (45) Consortium via the PRIDE (46) repository with the data set identifier PXD060135.

### Indirect immunofluorescence assay and immunoblotting

Parasites at the trophozoite stage and purified FVs were fixed with fixation solution containing 4% paraformaldehyde and 0.0075% glutaraldehyde in PBS for 30 min, followed by blocking with 10% fetal bovine serum. Immunostaining and Western blot analysis were performed as described elsewhere (43).

### Whole-genome sequencing data analysis and co-existing patterns of SNPs

The pairwise alignments of forward and reverse sequences were performed via ClustalW software and were refined manually to obtain a better consensus and mutation analysis. SNP data sets previously generated by Rawat et al. (2022) (47) were used to check for co-existing mutations present along with the PfKelch13 and PfCRT polymorphisms. Briefly, SNP data from Pf3K (Pilot Data Release 4) MalariaGEN (The Pf3K project, 2015) were used for the analysis (MalariaGEN *P. falciparum* Community Project, 2016) (24). A total of 2,517 genomic data sets (1,501 from Africa and 1,010 from Asia) were downloaded, and snpEFF version 4.3 was used to annotate the variant data (25). A binary matrix was created for the entire data set, where one signified the presence of a mutation, whereas 0 signified absence. From this matrix, our mutations of interest were filtered out, and the graphs were plotted in GraphPad (Prism10, Version 10.0.3, USA). To check for the significance of coassociation between the polymorphisms, we applied a χ independence test with a *P* value cut-off of 0.05 and a degree of freedom of 1 (standard for a 2 × 2 grid) (Table S1).

### Optimization of transporter genes for complementation in *S. cerevisiae* and site-directed mutagenesis

To investigate the effects of PfNT1 and PfMFR5, we conducted complementation studies in *S. cerevisiae BY4741*. The genes were sequence optimized and synthesized (Genescript, Singapore) for successful expression in yeast. The genes were subsequently cloned and inserted into the pGPD2 vector at the EcoRI and BamHI restriction sites. Following the synthesis of the plasmids, the *Pf*NT1 and *Pf*MFR5 constructs were transformed into *S. cerevisiae BY4741* cells via the lithium acetate transformation method, as described previously (48). Transformants were selected by plating onto synthetic complete medium lacking uracil, which serves as a selective medium.

The specific mutations were introduced into the pGPD2_NT1 and pGPD2_MFR5 constructs via the Q5 Site-Directed Mutagenesis Kit (New England Biolabs) according to the manufacturer’s protocols. The pGPD2_MFR5 and pGPD2_NT1 plasmids were amplified via custom-designed primers specific to the mutation sites (detailed in Table S2). After amplification, the PCR products were treated with kinase, ligase, and DpnI (KLD). The resulting KLD-treated plasmid mixture was then transformed into bacteria-competent cells via the Q5 Site-Directed Mutagenesis Kit. Sequencing was subsequently performed to confirm the successful integration of the desired mutation.

### Serial dilution spot assay and growth kinetics

BY4741 and plasmids containing *S. cerevisiae* strains were phenotypically characterized via a serial dilution spot assay (48). Briefly, the overnight-grown cells were normalized to an OD_600_ of 0.1 and further serially diluted 10-fold in 0.9% NaCl. Five microliters of serially diluted culture was then spotted on test plates. The plates were incubated at 30°C for 48 h, and growth was recorded by capturing images via a ChemiDoc XRS + Bio-Rad system.

Time course growth curve analysis of BY4741 mutant cells was performed in a 96-well plate via a liquid handling system (Tecon, Austria). For growth curve generation, the cells were first grown in appropriate media overnight, followed by subculturing in synthetic defined (SD) complete media with and without drugs with an initial OD_600_ of 0.2. Growth was monitored by recording the absorbance at 600 nm at 30 min to 24 h intervals. The absorbance values obtained were then plotted over time (48).

### *In silico* analysis of mutations conferring resistance against DHA in the PfNT1 and PfMFR5 proteins

To model the structure of PfNT1 and PfMFR5, their amino acid sequences were retrieved from the PlasmoDB database and submitted to the i-TASSER web server for homology modeling (49). This web server generates 3D models by threading the sequence through alignment of multiple templates in the protein data bank (PDB) database. The generated models were evaluated on the basis of the *C*-scores; we chose the model with the lowest *C*-score for further structural validation. The stereochemical and geometrical properties of the predicted model were validated via the PROCHECK tool (50). For docking studies, DHA molecules were downloaded from the PubChem database in SDF format and converted into PDB through PyMoL software. Molecular docking of DHA with PfNT1 and PfMFR5 was carried out via the Swiss dock web server (51), and the top binding poses were selected on the basis of cluster analysis, binding affinity, and key interactions with the active site pocket. Visualization and analysis of the protein‒ligand interactions were conducted via ChimeraX software. The WT model structure of PfNT1 and PfMFR5 was used for introduction of the mutation at the respective site via ChimeraX software.

## SUPPLEMENTAL MATERIAL

Supplemental figures aac.00293-25-s0001.pdf Fig. S1 to S9.

Supplemental material aac.00293-25-s0002.docx Legends for supplemental figures and tables.

Supplemental tables aac.00293-25-s0003.docx Tables S1 and S2.

Table S3 aac.00293-25-s0004.xlsx Proteome dataset of all the food vacuole proteins.

Table S4 aac.00293-25-s0005.xlsx Common FV proteins between Lamarque et al., 2008 and our study.

ASM does not own the copyrights to Supplemental Material that may be linked to, or accessed through, an article. The authors have granted ASM a non-exclusive, world-wide license to publish the Supplemental Material files. Please contact the corresponding author directly for reuse.
