# Supplementary material for: Role of novel mutations in food vacuole transporters beyond K13-mediated artemisinin resistance in Plasmodium falciparum
Source: Antimicrob Agents Chemother. 2025 Sep 30;69(11):e00293-25. doi: 10.1128/aac.00293-25 (PMC12587573; doi:10.1128/aac.00293-25)

Suppl. Figure -1

A Magnetic Isolation Method

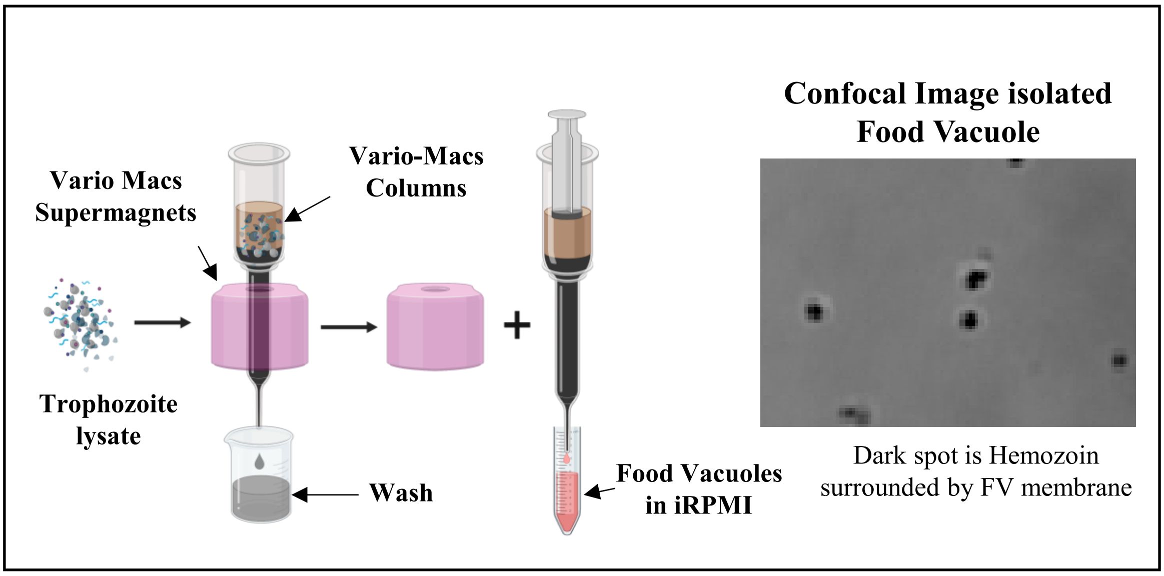

B Chemical Isolation Method

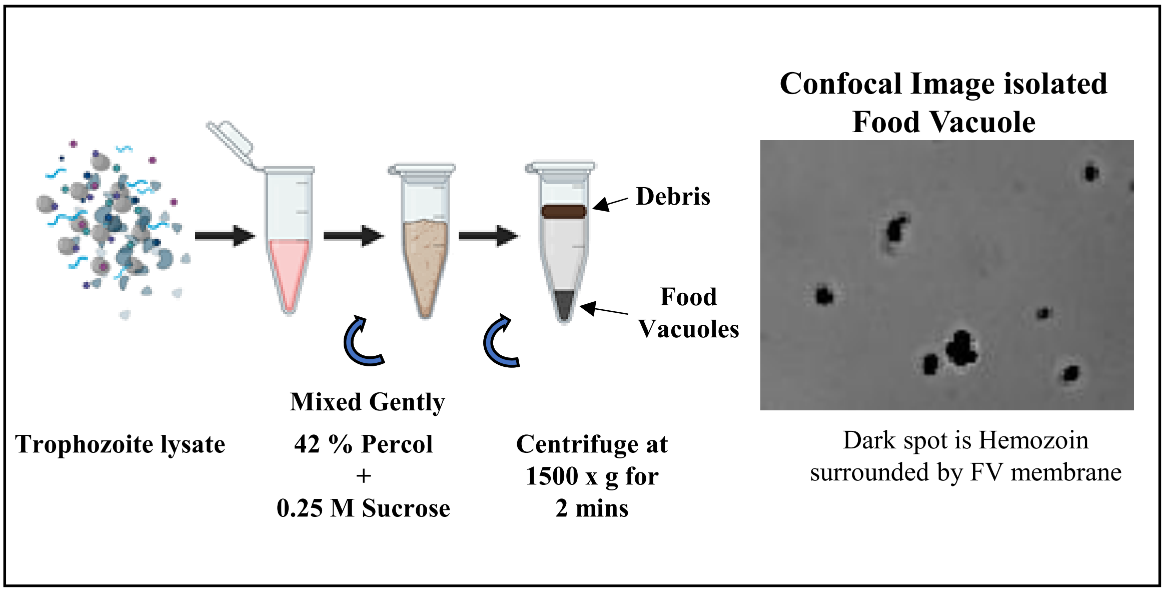

C Proteome comparison

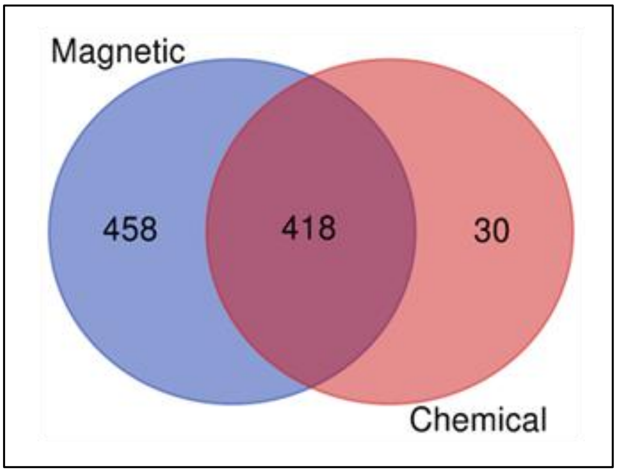

Suppl. Figure -2

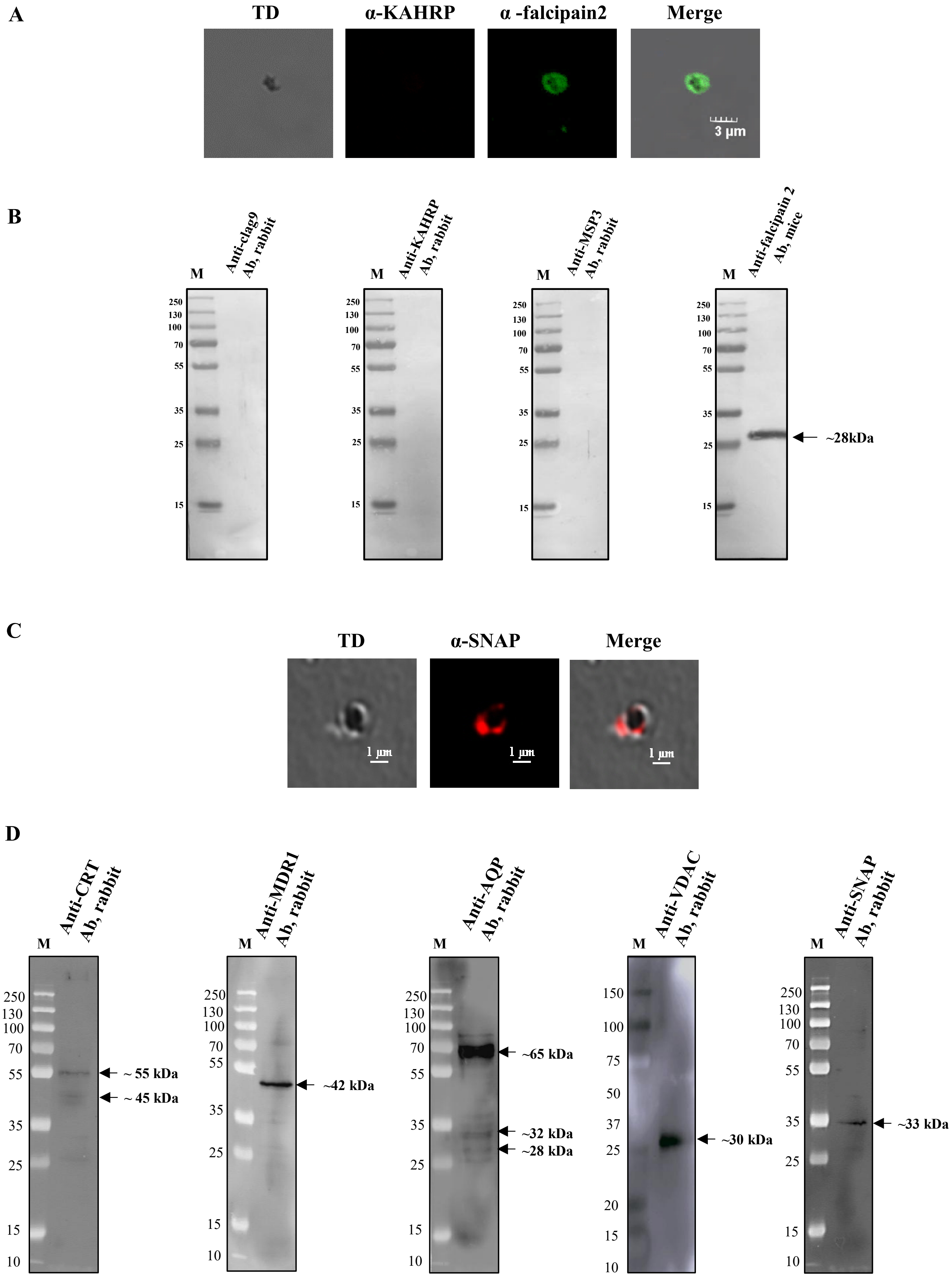

Suppl. Figure -3

A

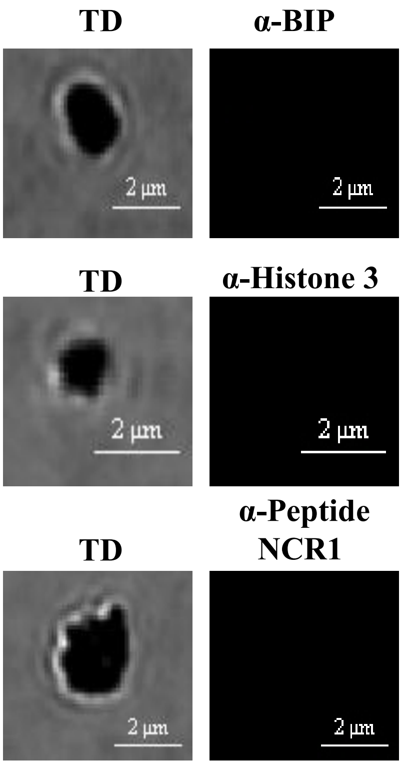

B

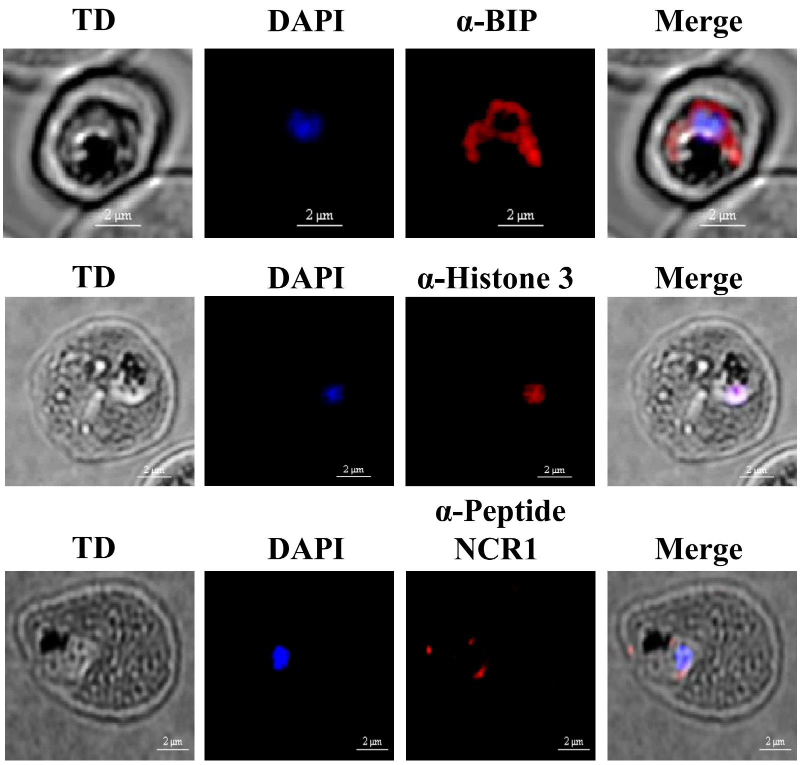

C

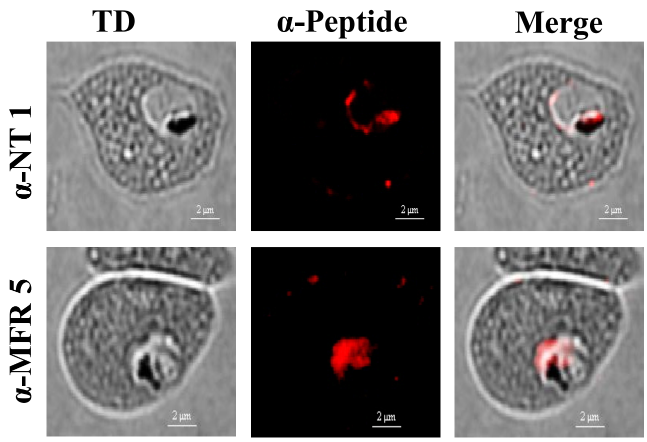

Suppl. Figure -4

A

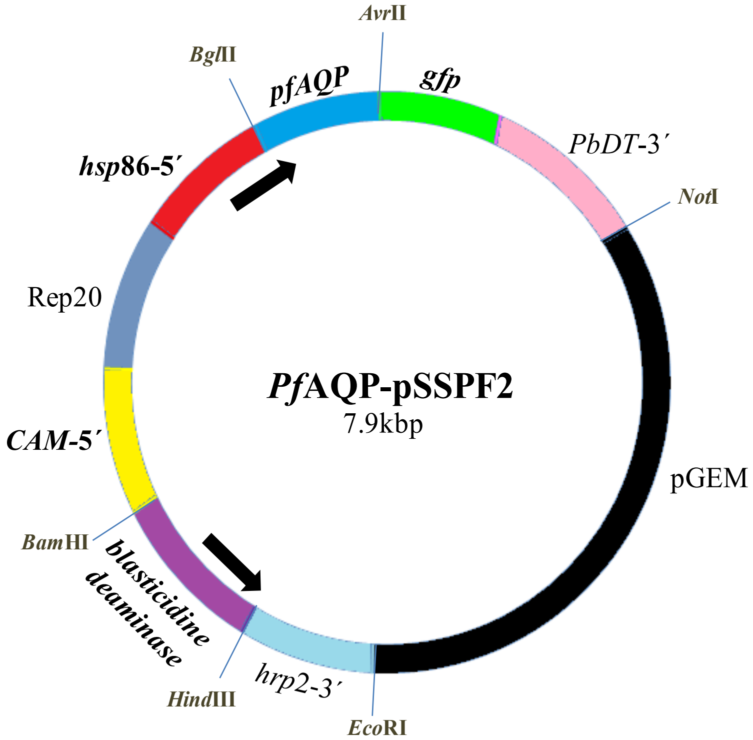

B

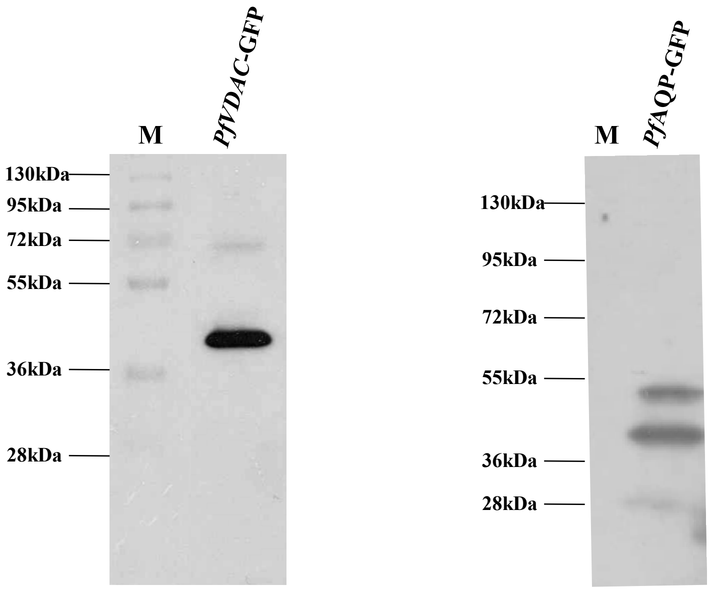

Suppl. Figure -5

A

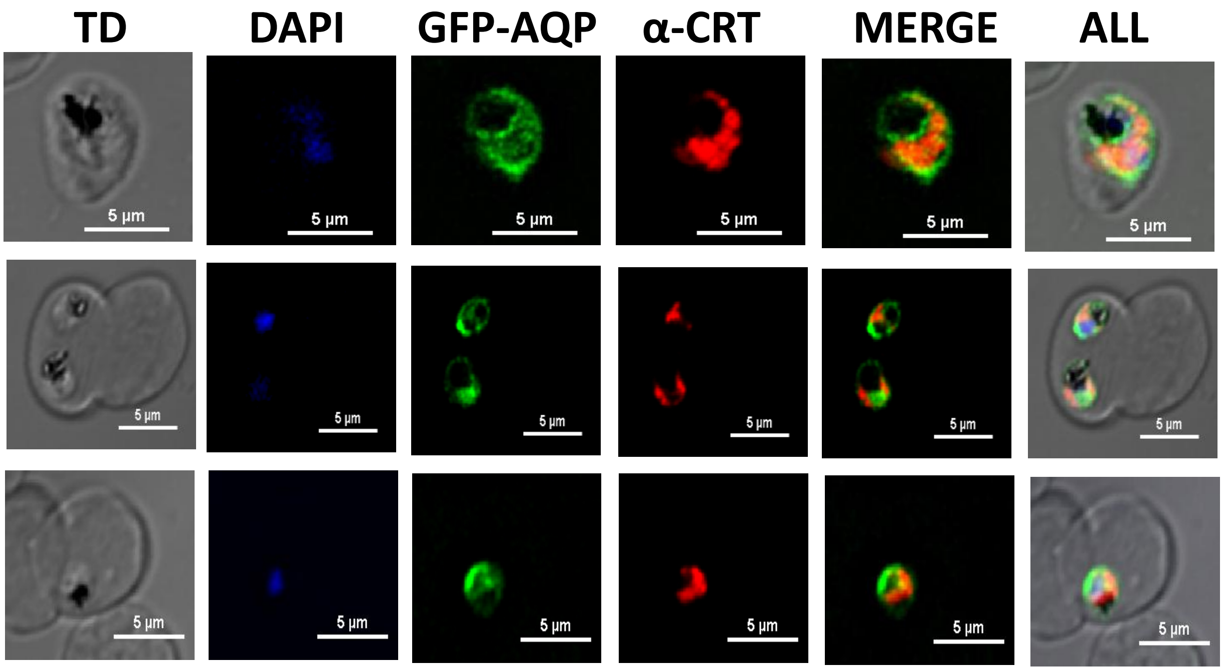

B

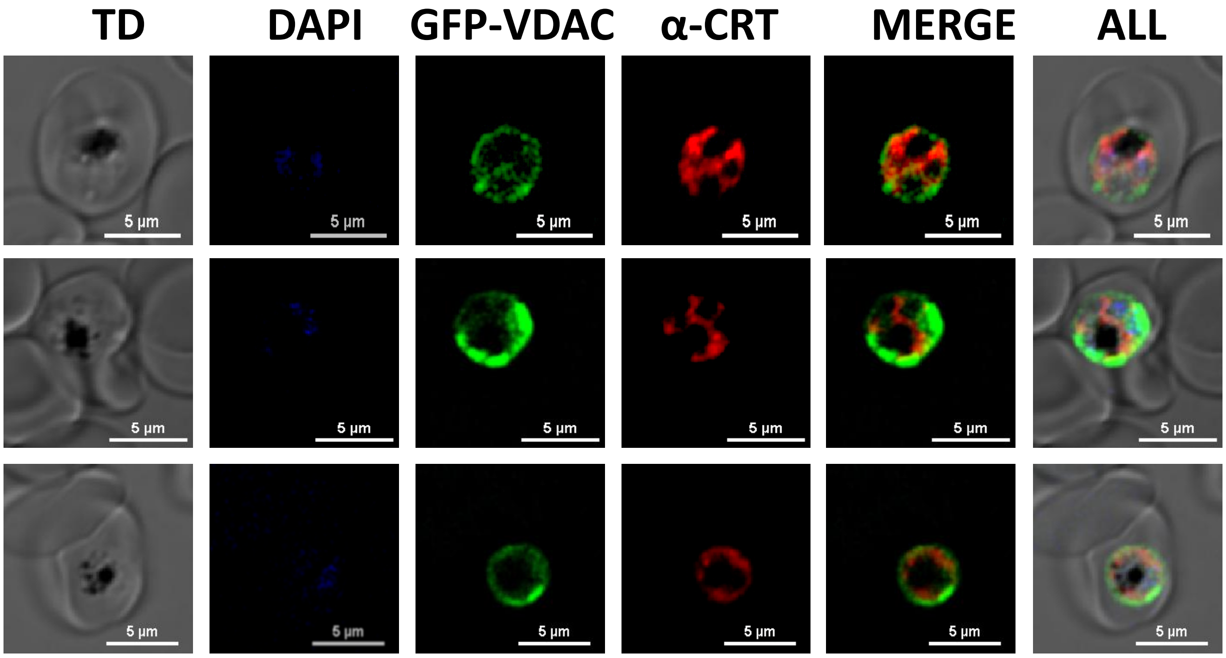

Suppl. Figure - 6

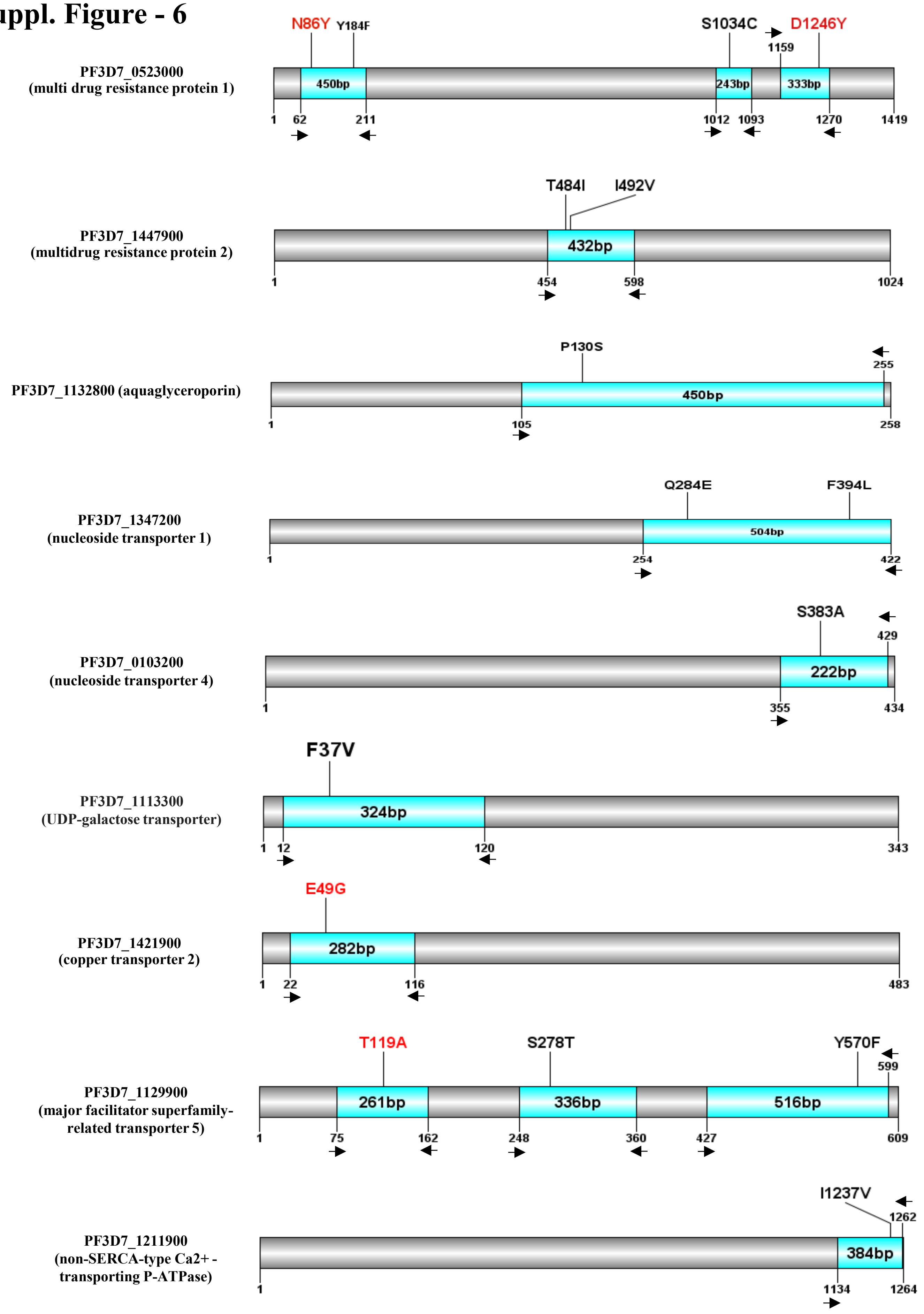

Suppl. Figure -7

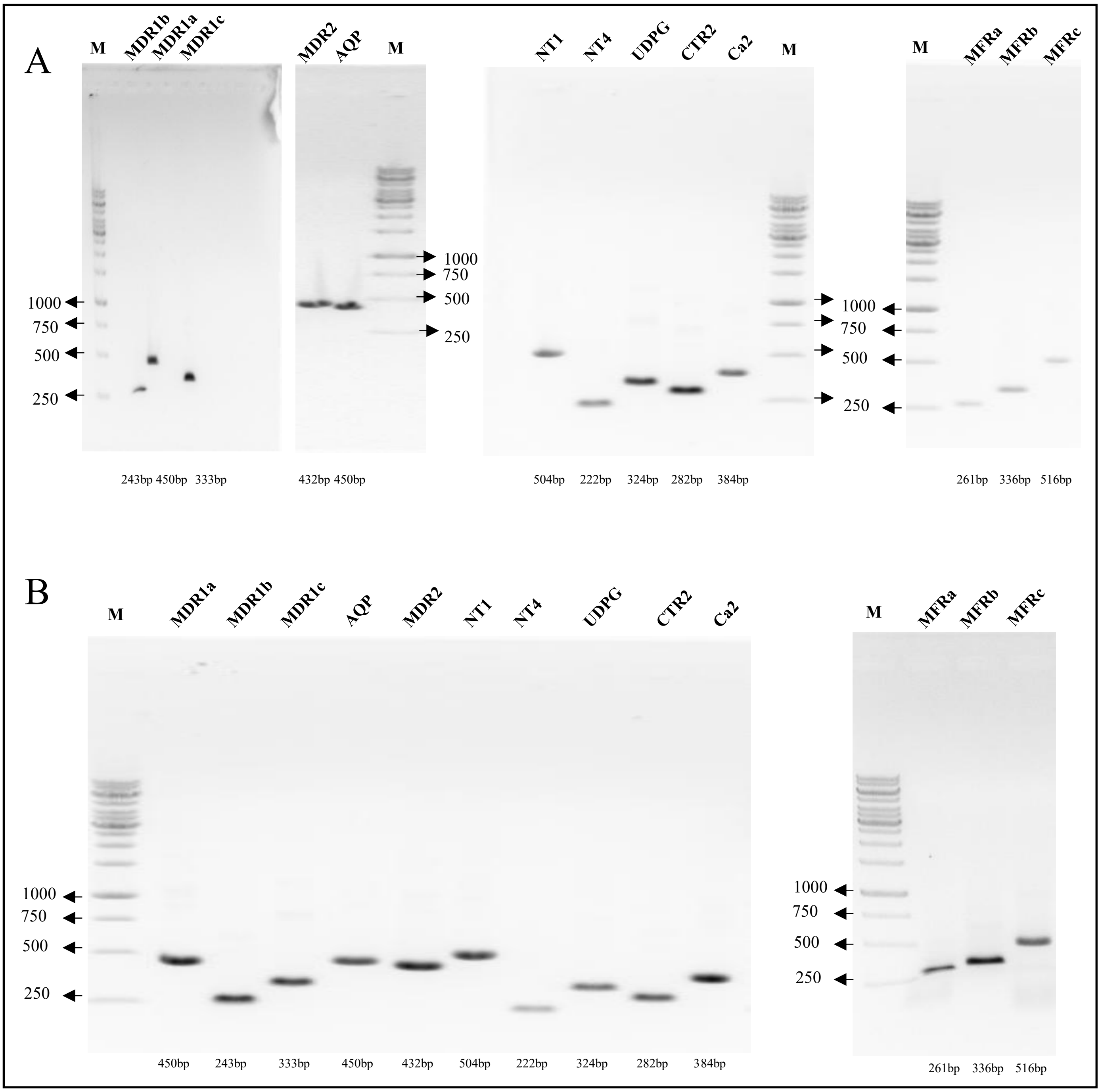

Suppl. Figure -8

Schematics of Protein folding of Food Vacuole Proteins

I. PF3D7_1447900 (Multidrug Resistance Protein 2)

Mutations identified in published genomes		Mutation identified in our study
3D7/DD2: S208N, G299D, N318del, F423Y, T484I	3D7/GB4: N318del	3D7/Indo: No change
3D7/7G8: S208N, F423Y, I492V	3D7/KH02: S208N, G299D, N318del, F423Y, T484I	3D7/1240: T484I

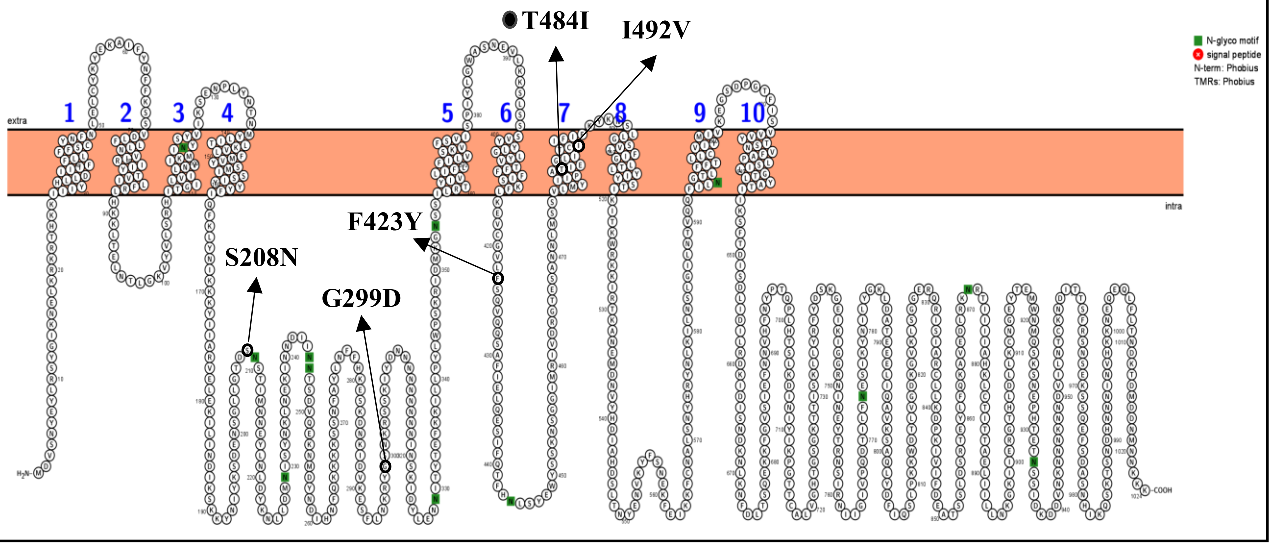

II. PF3D7_1129900 (Major Facilitator Superfamily-related Transporter 5)

Mutations identified in published genomes		Mutations Identified in our study
3D7/DD2: No change	3D7/GB4: A606V	3D7/Indo: No change
3D7/7G8: T119A,S278T,Y570F	3D7/KH02: No change	3D7/1240: *V549M

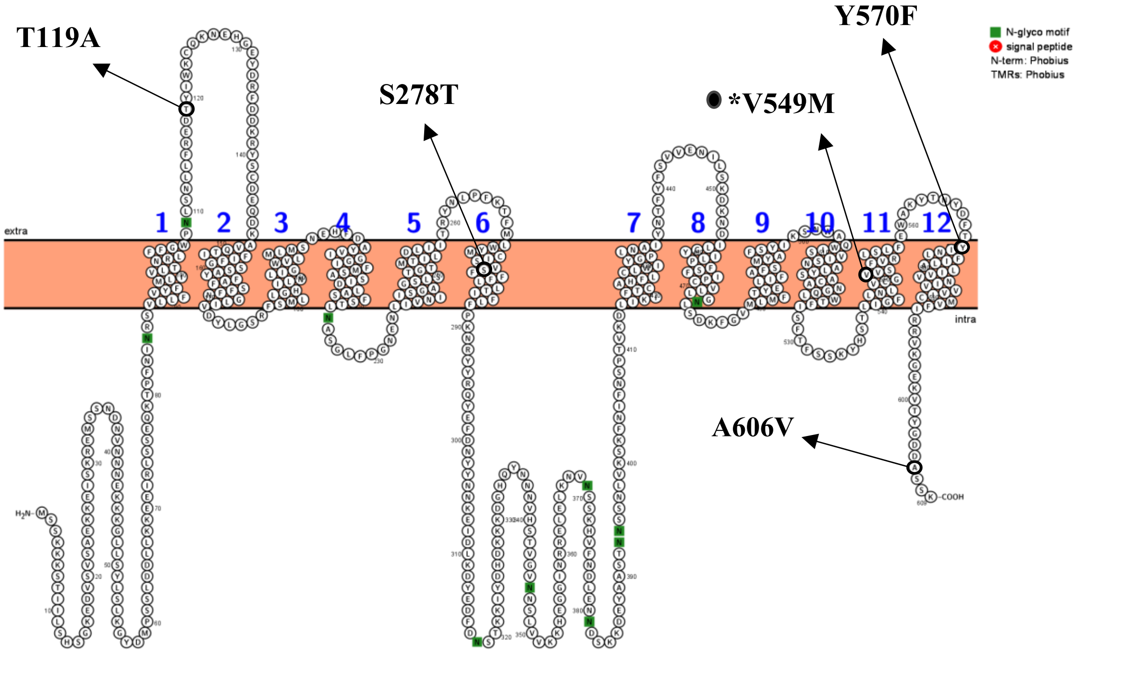

III. PF3D7_1347200 (Nucleoside Transporter 1)		
Mutations identified in published genomes		Mutations identified in our study
3D7/DD2: F394L	3D7/GB4: No change	3D7/Indo: F394L
3D7/7G8: Q284E	3D7/KH02: F394L	3D7/1240: F394L

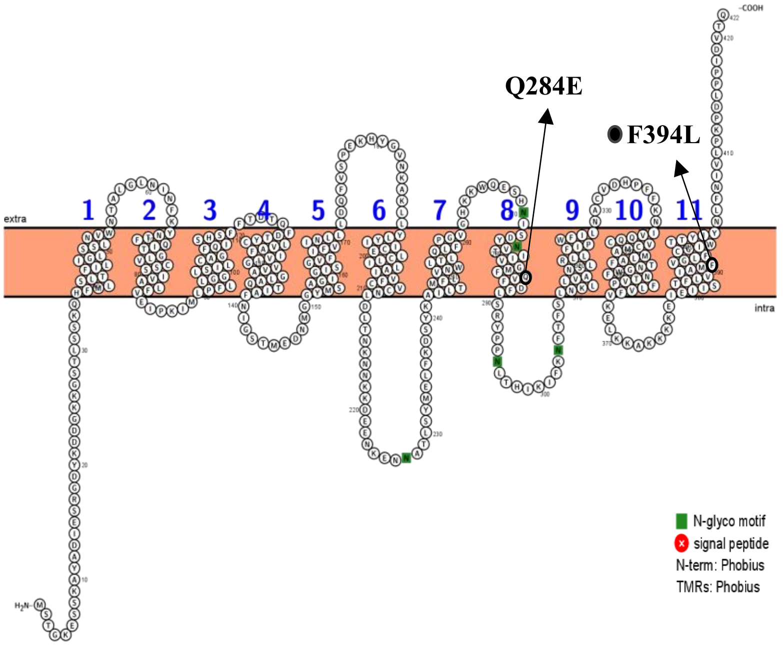

IV. PF3D7_1421900 (Copper Transporter 2)		
Mutations identified in published genomes		Mutations identified in our study
3D7/DD2: E49G	3D7/GB4: No change	3D7/Indo: E49G
3D7/7G8: No change	3D7/KH02: E49G	3D7/1240: No change

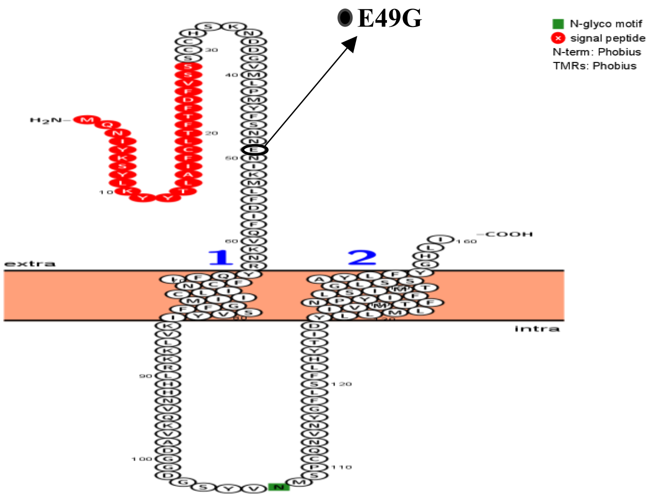

V. PF3D7_0523000 (Multidrug Resistance Protein 1)

Mutations in published genome		Mutations in our study
3D7/DD2: N86Y, N662del	3D7/GB4: N86Y, Y184F, D650N, N652D	3D7/Indo: Y184F, S1034C, N1042D
3D7/7G8: Y184F, N661del, S1034C, D1246Y,	3D7/KH02: Y184F, N659DEL, N660DEL, N661DEL, S784L	3D7/1240: Y184F

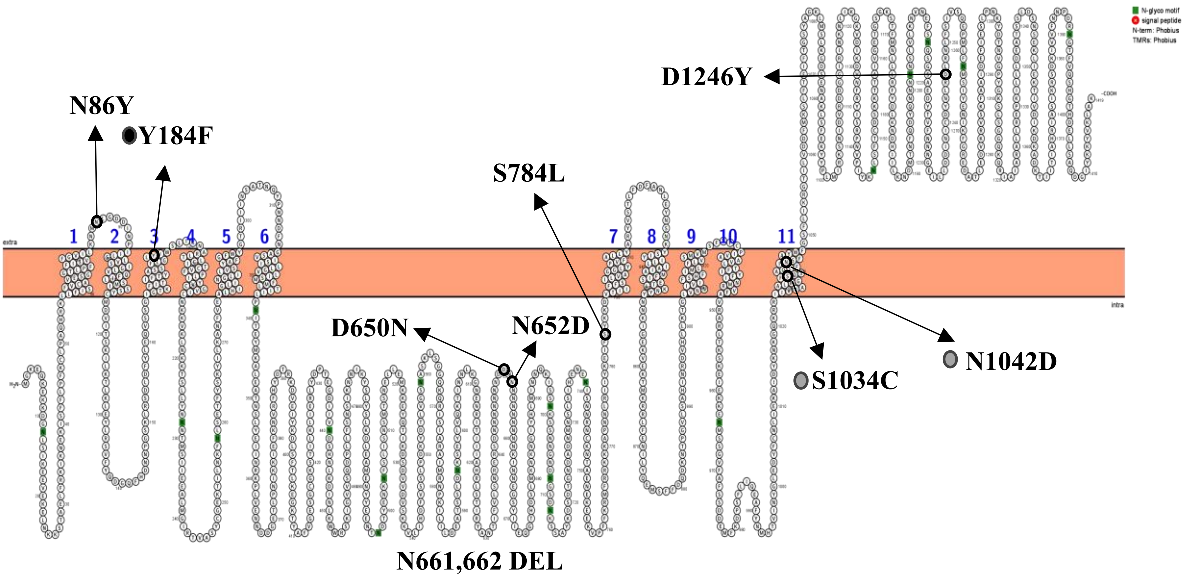

VI. PF3D7_1132800 (aquaglyceroporin)

Mutations in published genome		Mutations in our study
3D7/DD2: P130S	3D7/GB4: No change	3D7/Indo: P130S
3D7/7G8: No change	3D7/KH02: P130S	3D7/1240: No change

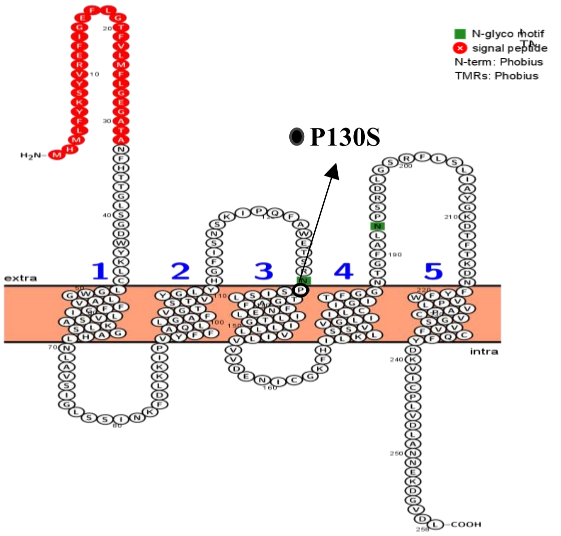

VII. PF3D7_0103200 (Nucleoside Transporter 4)

Mutations in published genome		Mutations in our study
3D7/DD2: S383A	3D7/GB4: No change	3D7/Indo: S383A
3D7/7G8: N209I	3D7/KH02: S383A	3D7/1240: S383A

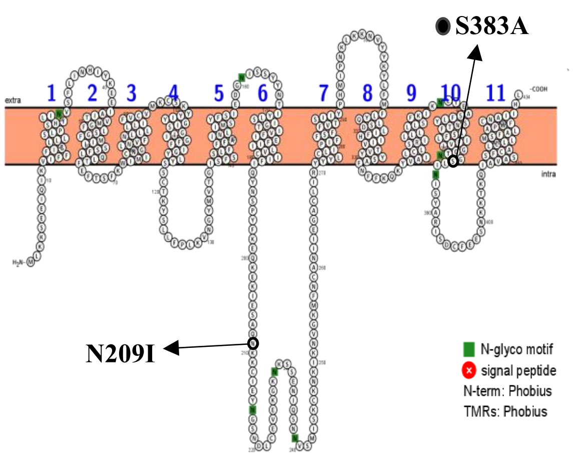

VIII. PF3D7_0824400 (Nucleoside Transporter 2)

3D7/DD2: S308T, S424T	3D7/GB4: No change
3D7/7G8: N53K, S308T, S424A, L470F, L524F, N525Y	3D7/KH02: S308T, S424T

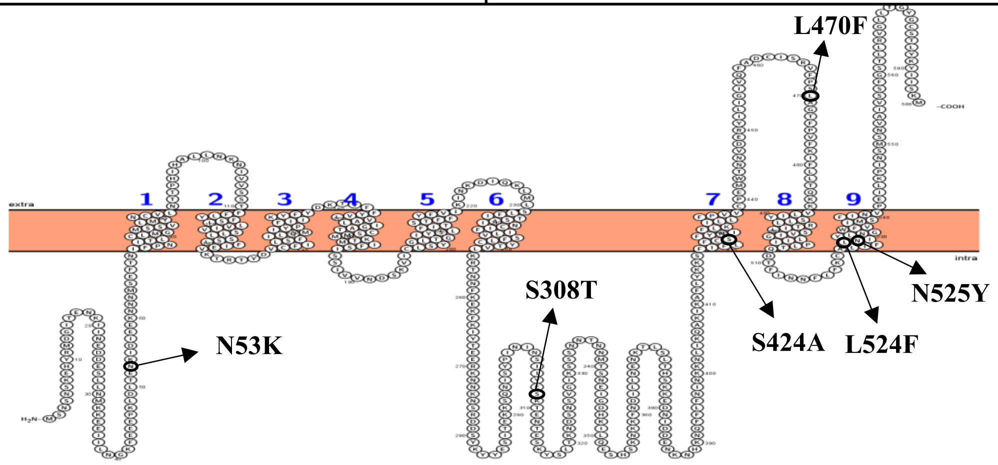

IX. PF3D7_0316600 (Formate-nitrite Transporter)

3D7/DD2: H159D	3D7/GB4: H159D
3D7/7G8: No change	3D7/KH02: H159D, E283G

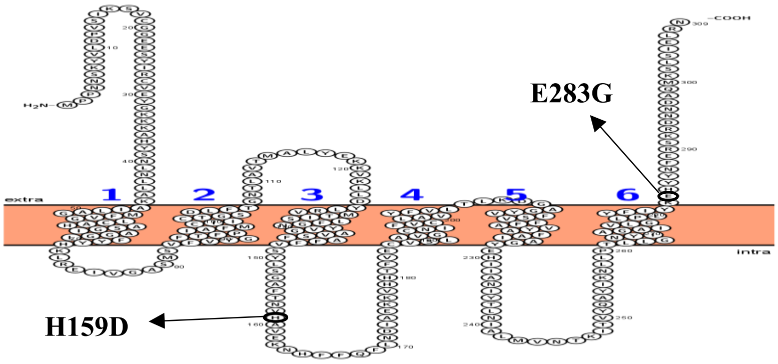

X. PF3D7_1036800 (Acetyl-CoA Transporter)

3D7/DD2: T459I	3D7/GB4: R104K, T459I
3D7/7G8: T459I	3D7/KH02: T459I

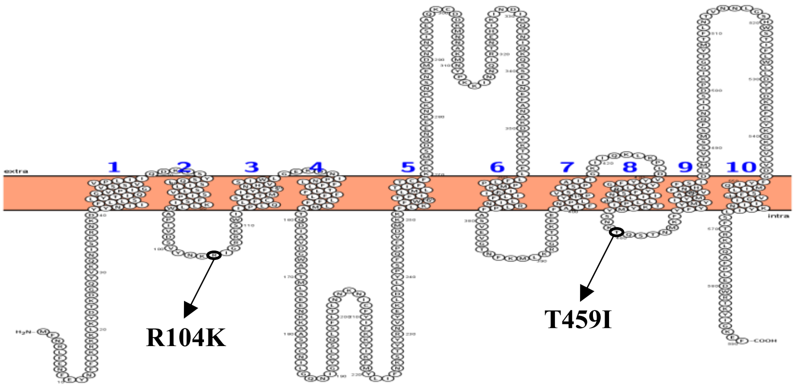

XI. PF3D7_1439000 (Copper Transporter 1)

3D7/DD2: S211L	3D7/GB4: No change
3D7/7G8: No change	3D7/KH02: No change

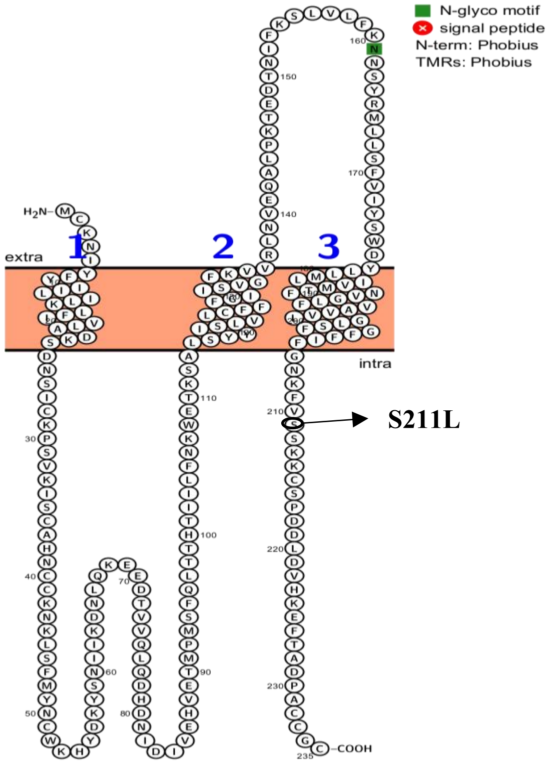

Suppl. Figure -9

A

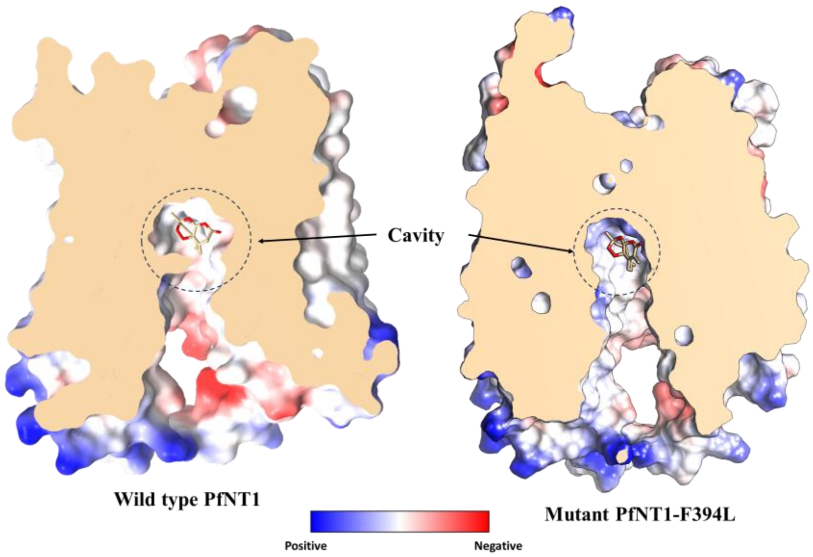

B

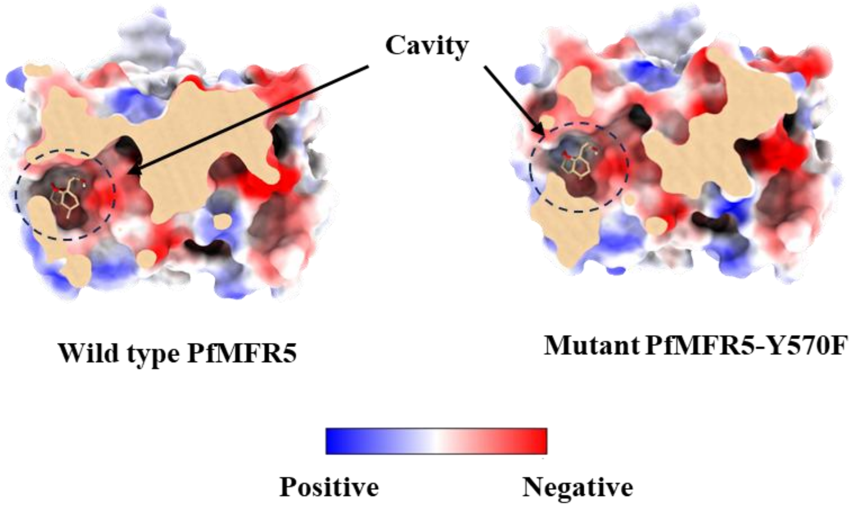

Supplement: Supplemental figures — Fig. S1 to S9. [file aac.00293-25-s0001.pdf]
