# Supplementary material for: Role of novel mutations in food vacuole transporters beyond K13-mediated artemisinin resistance in Plasmodium falciparum
Source: Antimicrob Agents Chemother. 2025 Sep 30;69(11):e00293-25. doi: 10.1128/aac.00293-25 (PMC12587573; doi:10.1128/aac.00293-25)
Supplement: Supplemental material — Legends for supplemental figures and tables. [file aac.00293-25-s0002.docx]

**Supplementary Figure Legends:**

**Suppl. Figure 1.** Schematic showing the steps involved in the purification of *P. falciparum* FVs via the (A) magnetic isolation method and (B) chemical isolation method. (C) Venn diagram showing the number of proteins identified by LC‒MS/MS analysis for FVs isolated by either chemical or magnetic methods and their overlap.

**Suppl. Figure 2.** Immunofluorescence and immunoblot analyses of purified FVs confirmed the isolation of FVs and the expression of transport proteins identified in the FV-enriched fraction. (A) Coimmunostaining of isolated FVs with anti-falcipain-2 and anti-KAHRP antibodies. Falcipain-2 is a known FV protein, while KAHRP is expressed on the surface of infected trophozoites. (B) Western blot analysis of purified FV extract using anti-falcipain-2 and anti-Clag9, anti-KAHRP and anti-MSP3 antibodies. A positive band of ~28 kDa was observed with anti-falcipain antibody, whereas no band was observed in blots stained with either anti-Clag9, anti-KAHRP, or anti-MSP-3 antibodies. (C) Immunostaining of isolated FVs via anti-PycSNAP, confirming the expression of the PfSNAP protein in parasite FVs. (D) Western blot analysis of purified FV extracts using anti-peptide PfCRT, PfMDR1, PfAQP, PfVDAC and PfSNAP antibodies confirmed the expression of the corresponding proteins in *Plasmodium* FVs.

**Suppl. Figure 3. Immunofluorescence-based validation of FV purity and subcellular localization of PfNT1 and PfMFR5.** (A) Immunostaining of isolated food vacuoles (FVs) with organelle-specific markers to assess contamination. FV preparations were negative for the endoplasmic reticulum (ER) chaperone BiP, the nuclear marker histone H3, and the plasma membrane marker Niemann-Pick Type C1 (NCR1), indicating minimal contamination from the ER, nucleus, or parasite plasma membrane (PPM). (B) Immunofluorescence assay on *Plasmodium falciparum* trophozoites using antibodies against BiP, histone H3, and NCR1, with DAPI staining to visualize nuclei. Positive staining in the parasite confirms the specificity of the antibodies, validating their use as compartment-specific controls. (C) Subcellular localization of PfNT1 and PfMFR5 in *P. falciparum* trophozoites. Immunostaining with peptide-specific antibodies revealed signal at both the food vacuole (FV) and the parasite plasma membrane (PPM), suggesting dual localization of these transporters.

**Suppl. Figure 4. Generation and confirmation of PfVDAC-and PfAQP-GFP fusion parasite lines.** (A) Schematic showing the vector map of the pSSPF2 construct used for generating the PfVDAC-GFP and PfAQP-GFP transgenic lines. (B) Western blot analysis of lysates from PfVDAC-GFP- and PfAQP-GFP-tagged lines using α-GFP rabbit antibodies. M denotes a known molecular weight marker.

**Suppl. Figure 5.** *Plasmodium falciparum* transgenic lines expressing the (A) PfAQP-GFP and (B) PfVDAC-GFP confirmed the expression of the PfAQP and PfVDAC proteins in FVs at the trophozoite stage.

**Suppl. Figure 6. Schematics of nine *Plasmodium falciparum* food vacuole transport proteins used to depict the amplified regions**. The blue regions indicate the amplified fragment for each gene. The primers (forward and reverse) are indicated by arrows. Lines depict the observed point mutations (red indicates mutations in extracellular domains, and black indicates mutations in TM domains).

**Suppl. Figure 7.** Ethylene bromide-stained agarose gel showing amplified fragments of target *Plasmodium falciparum* food vacuole transporter/channel genes. Each band was excised, and DNA was extracted and sequenced.

**Suppl. Figure 8. *Plasmodium falciparum* food vacuole transport protein mutations in different isolates.** Schematic representation of the Pf MDR2 (i), PfMFR5 (ii), PfNT1 (iii), PfCTR2 (iv), Pf MDR1 (v), PfAQP (vi), PfNT4 (vii), PfNT2 (viii), PfFNT (ix), PfACT (x) and PfCTR1 (xi) proteins. The mutation sites are circled, and arrows indicate the positions and mutations. Indicates mutations identified in this study. * Indicates novel mutations identified in this study.

**Suppl. Figure 9.** (A) The dotted circle represents the ligand binding cavity of wild-type PfNT1 and mutant PfNT1-F394L. The mutant PfNT1-F394L exhibited a shift in the local electrostatic environment from neutral in the wild type to a positive charge. (B) The dotted circle represents the ligand binding cavity of wild-type PfMFR5 and the mutant PfMFR5-Y570F. Compared with its wild-type form, the mutant PfMFR5-Y570F showed no changes in electrostatic potential.

**Supplementary Tables**

**Suppl. Table 1.** Chi square test statistics for comparing WT and K13 mutant instances of candidate genes.

**Suppl. Table 2.** Details of primers for site-directed mutagenesis in codon-optimized pGPD2NT1 and pGPD2MFR5 plasmids

**Suppl. Table 3**. List of protein hits identified by LC/MS analysis of FV isolated by Magnetic and Chemical method

**Suppl. Table 4.** List of common proteins identified in Lamarque et.al., 2008 and our study
