# Supplementary material for: Role of novel mutations in food vacuole transporters beyond K13-mediated artemisinin resistance in Plasmodium falciparum
Source: Antimicrob Agents Chemother. 2025 Sep 30;69(11):e00293-25. doi: 10.1128/aac.00293-25 (PMC12587573; doi:10.1128/aac.00293-25)
Supplement: Supplemental tables — Tables S1 and S2. [file aac.00293-25-s0003.docx]

**Suppl. Table 1. chi square test statistics for comparing WT and K13 mutant instances of candidate genes.**

| **PF3D7_0709000_N326S** |  | **K13 wt** | **K13 mut** |  | The chi-square statistic is 987.325. The p-value is < .0001. The result is significant at p < .05. |
| --- | --- | --- | --- | --- | --- |
|  | N326N (wt) | 1812 | 23 |  |  |
|  | N326S (mut) | 332 | 350 |  |  |
|  |  |  |  |  |  |
| **PF3D7_0709000_I356T** |  | **K13 wt** | **K13 mut** |  | The chi-square statistic is 745.151. The p-value is < .0001. The result is significant at p < .05. |
|  | I356I (wt) | 1643 | 15 |  |  |
|  | I356T (mut) | 501 | 358 |  |  |
|  |  |  |  |  |  |
| **PF3D7_1347200_F394L** |  | **K13 wt** | **K13 mut** |  | The chi-square statistic is 1049.053. The p-value is < .0001. The result is significant at p < .05. |
|  | F394F (wt) | 1877 | 38 |  |  |
|  | F394L (mut) | 266 | 336 |  |  |
|  |  |  |  |  |  |
| **PF3D7_1421900_E49G** |  | **K13 wt** | **K13 mut** |  | The chi-square statistic is 189.515. The p-value is < .0001. The result is significant at p < .05. |
|  | E49E (wt) | 1576 | 140 |  |  |
|  | E49G (mut) | 568 | 233 |  |  |
|  |  |  |  |  |  |
| **PF3D7_1447900_G299D** |  | **K13 wt** | **K13 mut** |  | The chi-square statistic is 270.236. The p-value is < .0001. The result is significant at p < .05. |
|  | G299G (wt) | 1571 | 112 |  |  |
|  | G299D (mut) | 572 | 262 |  |  |
|  |  |  |  |  |  |
| **PF3D7_1447900_T484I** |  | **K13 wt** | **K13 mut** |  | The chi-square statistic is 225.408. The p-value is < .0001. The result is significant at p < .05. |
|  | T484T (wt) | 1713 | 161 |  |  |
|  | T484I (mut) | 431 | 212 |  |  |

**Suppl. Table 2. Details of primers for site directed mutagenesis in codon optimized pGPD2NT1 and pGPD2MFR5 plasmids.**

| Primer Name | Sequence | Purpose |
| --- | --- | --- |
| NT1_F394L_FP | CGTTGGTTTGctgTGTGGTATCTG | Forward primer for site-directed mutagenesis of pGPD2_NT1 |
| NT1_F394L_RP | AACATTGCAATAACCAAAAATG | Reverse primer for site-directed mutagenesis of pGPD2_NT1 |
| MFR5_S278T_FP | TATCTCTGTTaccTTTTTCTTTTTGTTAACAATTTTC | Forward primer for site-directed mutagenesis of pGPD2_MFR5 |
| MFR5_S278T_RP | CACATGTACCACAACATAAAAG | Reverse primer for site-directed mutagenesis of pGPD2_MFR5 |
| MFR5_Y570F_FP | CGATTTCACTtttATTAATTTGTTTATTTTGG | Forward primer for site-directed mutagenesis of pGPD2_MFR5 |
| MFR5_Y570F_RP | TAGTTTGTGTACTTAGCC | Reverse primer for site-directed mutagenesis of pGPD2_MFR5 |
